# Sex differentially affects pro-inflammatory cell subsets in adipose tissue depots in a diet induced obesity model

**DOI:** 10.1186/s13293-024-00677-1

**Published:** 2024-12-18

**Authors:** Lisa T. Schuetz, Gayel Duran, Paulien Baeten, Daphne Lintsen, Doryssa Hermans, Sarah Chenine, Janne Verreycken, Tim Vanmierlo, Kristiaan Wouters, Bieke Broux

**Affiliations:** 1 Neuro-Immune Connections and Repair Lab, Department of Immunology and Infection, Biomedical Research Institute, Hasselt University, Diepenbeek, Belgium; 2 CARIM-School of Cardiovascular Diseases, Maastricht University, Maastricht, Netherlands; 3 Internal Medicine, Maastricht University, Maastricht, Netherlands; 4 University MS Center, Campus Diepenbeek, Diepenbeek, Belgium; 5 MHeNs-Mental Health and Neuroscience Institute, Maastricht University, Maastricht, Netherlands

**Keywords:** Diet-induced obesity, Sex, Inflammation, Adipose tissue, Inflammasome

## Abstract

Obesity is a growing pandemic that increases the risk for cardiovascular diseases, type 2 diabetes, and particularly in women also the risk of cancer and neurodegenerative disorders such as dementia and multiple sclerosis. Preclinical studies on obesity focus on male mice as they gain bodyweight faster and show a clear pro-inflammatory phenotype. Here, using male and female mice, we induced obesity by feeding a high fat diet (HFD), and compared adipose tissue (AT) inflammation at the same adiposity stage (% AT/bodyweight) between both sexes. Doing so, we identified that female mice show an increase in the number of pro-inflammatory immune cells in the visceral AT at a lower adiposity stage than male mice, but the effect of HFD is diminished with higher adiposity. Interestingly, only female mice showed an increase in immune cells in the subcutaneous AT after HFD feeding. Nonetheless, we found that pro-inflammatory cytokines in blood plasma mirror the inflammatory stage of the visceral AT in both male and female mice. Uniquely in male mice, myeloid cells in the visceral AT showed a higher inflammasome activation upon HFD. In summary, we showed that adiposity differentially affects immune cells in fat depots based on sex.

**Supplementary Information:**

The online version contains supplementary material available at 10.1186/s13293-024-00677-1.

## Highlights

- While female mice gain weight more slowly than males, they can still reach comparable adipose tissue-to-body weight ratios.
- In male mice, a high-fat diet primarily induces immune cell changes in visceral fat, whereas in female mice, these changes are also seen in subcutaneous fat.
- Our obesity model shows that NLRP3 inflammasome activation occurs exclusively in the myeloid cells of visceral fat in male mice.

**Supplementary Information:**

The online version contains supplementary material available at 10.1186/s13293-024-00677-1.

## Abstract (plain language summary)

The prevalence of obesity is rising, and it is a known risk factor for conditions such as cardiovascular disease and diabetes. One mechanism through which obesity heightens these risks is by promoting activation of the immune system. To investigate this, researchers often use male mice in studies because they tend to gain weight more rapidly than females. However, in humans, the health impacts of obesity can vary by sex, suggesting that sex-based differences may also be relevant in mouse models.

In our study, we found that while male mice gain weight more quickly, both male and female mice can achieve similar fat-to-body weight ratios if given adequate time. Interestingly, inflammation was distributed differently across fat depots depending on sex. In female mice, inflammation was more pronounced in subcutaneous fat (under the skin), whereas in male mice, obesity-induced inflammation was more significant in visceral fat (around the organs). Moreover, only male mice exhibited increased activation of the inflammasome—a protein complex triggered by cellular stress—in specific immune cells that serve as the first line of defense against infections.

In conclusion, we demonstrate that sex influences how obesity drives inflammation in different fat depots in mice. This observation aligns with human studies showing differences in body composition between men and women. Recognizing the role of sex in obesity-related inflammation could improve the design of future mouse studies targeting obesity and its associated inflammatory responses.

**Supplementary Information:**

The online version contains supplementary material available at 10.1186/s13293-024-00677-1.

## Introduction

Obesity is a major worldwide health problem that is defined by excessive fat accumulation and is recognised as a chronic disease [1, 2]. It has a multifactorial origin, however, a chronic energy imbalance due to a caloric surplus is a major driver [3]. Since 1975, obesity prevalence nearly tripled and according to the World Health Organization (WHO), 13% of adults had a body mass index (BMI) ≥ 30 kg/m^2^ and around 7% of children and adolescents aged 5–19 fit the obesity criteria in 2016. As obesity is a major risk factor for non-communicable diseases like cardiovascular diseases, type 2 diabetes and musculoskeletal disorders, the disease burden is extensive [1]. This high disease burden and the comorbidities that arise due to obesity, highlight the demand for better understanding the changes that occur due to fat accumulation. As a result, the number of published preclinical studies increased over the last decade, however, most studies focus on male mouse models [4–6]. The underlying reason is that male obesity models are faster to develop, more stable, and lead to a more pronounced metabolic dysregulation and a clear inflammatory state [7, 8]. However, in humans obesity is more prevalent in women and comorbidities differ based on sex, as in women the risk for cancer and neurodegenerative disorders such as dementia and multiple sclerosis is higher than in men with obesity [2, 7–10]. This emphasizes the importance of investigating how sex affects obesity related complications. Therefore, we aim to improve the knowledge on how sex affects inflammatory changes in a preclinical diet-induced obesity (DIO) model.

In the white adipose tissue (AT), obesity leads to a pro-inflammatory environment, especially in the visceral AT (vAT). In the obesity-expanded AT, macrophages gain a pro-inflammatory phenotype and secrete a variety of cytokines in response to obesity. Furthermore, they produce chemokines that attract additional immune cells from the circulation, such as monocytes, neutrophils, CD8^+^ and CD4^+^ T cells. In turn, these immune cells also secrete pro-inflammatory cytokines including interleukin (IL)-6, interferon (IFN)-γ or tumor necrosis factor (TNF)-α, leading to a vicious cycle of chronic vAT inflammation [11–13]. Interestingly, within this complex interplay of immune cells in the AT, it has recently been shown that obesity-memory is retained within the CD4^+^ T cell subset. More specifically, adoptive transfer of T helper (Th) cells from male obese mice promotes weight regain in male recipient mice [14]. In addition, several studies reported that nucleotide-binding domain, leucine-rich-containing family, pyrin domain-containing-3 (NLRP3) inflammasome activation is an important player in AT inflammation, as it contributes to obesity-induced inflammation and is linked to insulin resistance and atherosclerosis in mice and humans [10, 15–19]. NLRP3 inflammasome activation typically involves the specking of apoptosis-associated speck-like protein containing a C-terminal caspase recruitment domain (ASC), and leads to the release of the pro-inflammatory cytokines IL-1β and IL-18, contributing to the chronic inflammation in AT [18, 20, 21]. Inflammasomes are seen as a part of the innate immune system, but in recent years their role in the adaptive immune system (e.g. in T cells) became evident, although no information is available yet on this mechanism in obesity [22].

To understand how sex affects immune cells in the adipose tissue, we aimed to investigate how sex interacts with the effect of obesity using a preclinical DIO model induced by HFD. We observed effects on immune cell populations in the AT, including T cells, B cells and myeloid cells. In male mice, HFD led to a steady increase in pro-inflammatory cell numbers in the vAT, concurrent with increasing adiposity, and inflammasome activation in myeloid cells due to DIO. Contrarily, in female mice, the initial increase in their immune cell numbers ceased at a higher adiposity stage (AS). However, only in female mice, a higher number of immune cells was found in subcutaneous AT (scAT) due to DIO. Taken together, we show sex-driven differences in HFD-induced AT inflammation, warranting careful consideration about the use of preclinical models for obesity research and subsequent interpretations.

## Material and methods

### Mice

Animal experiments were performed using in house homozygous bred R26-CAG-ASC-citrine mice (on C57Bl/6J background), a reporter mouse model for inflammasome assembly, (Jackson Laboratory, #030744) [23]. As reported in the original paper, these mice do not show increased inflammation, as the construct results in functional ectopic expression, without increased inflammasome activation [23]. 32 male and 34 female mice were socially housed in an accredited animal facility under a 12 h light–dark cycle with cage enrichment and ad libitum access to food and water. All procedures were conducted in accordance with the EU directive 2010/63/EU and with prior approval from the Hasselt University Ethics Committee for Animal Experiments (PLL number 202220).

**Fig. 1:**
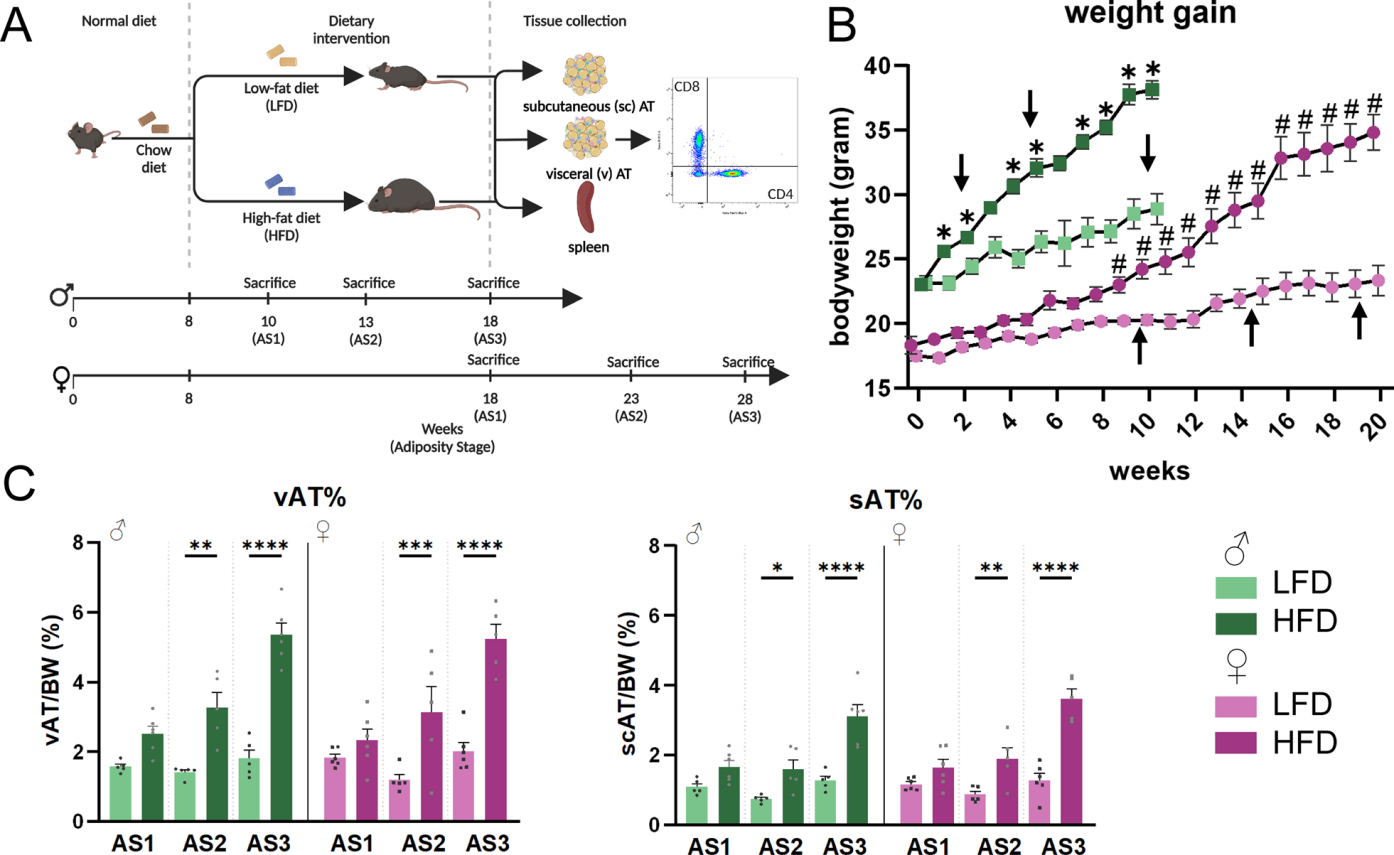
Comparable adiposity develops at different timepoints in male and female mice. **A** Experimental setup (created with BioRender). **B** Bodyweight (gram) was measured weekly from the start of the diet (week 0) until the time of sacrifice (arrows, AS1-3). Male mice gained weight earlier on HFD than female mice. As seen by the SEM, weight gain also varied less between male mice than within female mice. p ≤ 0.05*. **C**, **D** Relative vAT and scAT weight at sacrifice. (n = 6/♂ AS1 HFD, AS3 HFD, ♀ AS1 HFD, LFD, AS3 LFD; n = 5/♂ AS1 LFD, AS2 HFD, LFD, AS3 LFD, ♀ AS2 HFD, LFD, AS3 HFD); p ≤ 0.05*, p ≤ 0.005**, p ≤ 0.001***, p ≤ 0.0001****

Mice received high fat diet (60% fat from lard, HFD, EF D12492, Bio-Services B.V.) or low fat diet (LFD, EF D12450J, Bio-Services B.V.) from the age of approximately 8 weeks for up to 20 weeks. The composition of the diets can be found in Table S1. Before the study, a pilot experiment was done with male and female mice on the diet for 10 and 20 weeks. Based on weight gain, and sex differences observed in both diet durations, we chose the timepoints for male and female mice (Fig. 1) that led to comparable adiposity. Mice were allocated to a group by matching their bodyweight at the start of the dietary intervention (n = 5–6). Mice were weighed weekly from the start of the diet. For post-mortem analyses, mice were sacrificed by sedation with sodium pentobarbital (200 mg/kg bodyweight) followed by transcardial perfusion with PBS/Heparin to harvest fat depots, spleen, and blood plasma. Tissues were stored on ice until cell isolation.

### Tissue isolation

#### Adipose tissue

Visceral perigonadal and subcutaneous inguinal fat pads were harvested (carefully excluding the lymph nodes), weighed, and subsequently minced and incubated with 3.4 mg Collagenase I (Sigma), 0.63 mg Collagenase XI (Sigma), and 0.4 mg DNAse I (Roche Diagnostics GmbH) in 5 ml RPMI-25 mM HEPES (Gibco) at 37 °C for 1 h. The dissolved tissue was filtered through a 70 µm cell strainer to obtain a single cell suspension with RPMI+ 10% fetal bovine serum (FBS). The suspension was centrifuged at 500*g* for 10 min at 4 °C. The supernatant including the floating adipocyte fraction was removed and the pellet was resuspended in PBS before another centrifugation step. Erythrocytes were lysed with 0.83% ammonium chloride for 1 min. Cells were washed and centrifuged. Cells were counted with a Moxi™ Z cell counter (Orflo Technologies) by counting all events (live and dead) that had a diameter ≥ 5 µm. The whole stromal vascular fraction (SVF) was resuspended in 200 µl PBS and used for flow cytometry.

#### Spleen

Spleens were placed in 3 ml RPMI with 0.5% Penicillin–Streptomycin (Thermo Fisher) and homogenized with a piston through a 70 µm cell strainer to obtain a single cell suspension. Cells were centrifuged at 250*g* for 10 min. Pellets were incubated for 4 min with 1 ml 0.83% ammonium chloride to remove erythrocytes. The cell suspension was centrifuged and filtered through a 70 µm cell strainer and washed. After a last centrifugation the cell suspension was resuspended in 4 ml RPMI with 0.5% Penicillin–Streptomycin and 10% FBS. Cells/ml were counted with the Moxi™ Z cell counter. One million cells were used for flow cytometry.

#### Blood plasma

Prior to perfusion, the right heart ventricle was punctured with a heparin coated syringe to obtain blood. Samples were stored on ice until centrifugation at 400*g* for 10 min (4 °C). The upper phase was transferred and centrifuged at 1500 g for 15 min (4 °C). Plasma was stored until further use at − 80 °C.

### Flow cytometry

Live/Dead staining (1/1000, Zombie NIR, Biolegend) was performed prior to blocking in 10% normal rat serum in PBS in a 96 well v-plate. Samples were incubated for 30 min with 1/100 CD3 (BV605, CAT# 100237), 1/50 CCR6 (PE-Cy7, CAT# 129816), and 1/200 CD19 (BV650, CAT# 115541), CD4 (Pacific Blue, CAT# 100428), CD8 (BV510, CAT# 100751), CD45 (Alexa Fluor 700, CAT# 103128), CD11b (PerCP-Cy5.5, CAT# 101228), CXCR3 (PE, CAT# 126505) and CD25 (APC, CAT# 102012) (all Biolegend) in PBS with 10% FBS and 1% sodium azide. Samples were acquired on BD LSR Fortessa™ (BD Biosciences) and analysed using FlowJo™ 10.8.1 (BD Biosciences). Absolute cell numbers for different subsets were calculated by multiplying the % of the FSC-A subset gate (Fig. S1; based on the Moxi™ Z gate), with the Moxi™ Z-counted cells prior to the FC staining. Inflammasome activation is based on ASC specking assessed by flow cytometry as previously described in [20]. The full gating strategy can be found in Fig. S1.

### LEGENDplex™

13 cytokines associated with inflammation were measured in blood plasma with a LEGENDplex™ mouse inflammation panel (Cat# 740,150, Biolegend) according to the manufacturer’s protocol. Briefly, samples were diluted twofold with assay buffer and incubated with capture beads in a 96 well v-plate. Biotinylated detection antibodies and streptavidin–phycoerythrin were added to assess fluorescent signal intensities for each analyte-specific population with BD LSR Fortessa™. Concentrations were determined based on a standard curve using LEGENDplex™ Data Analysis Software Suite. In case a value was under the detection limit, a value of zero was used during analysis.

### Inclusion and exclusion criteria

Mice were excluded from the analysis if other inflammatory processes like infections/wounds were observed at sacrifice, or if HFD did not lead to the expected weight gain. In addition, despite careful dissection, three scAT samples were excluded due to contamination with lymph nodes. Six more samples were excluded as a result of technical issues that led to low cell numbers in the adaptive immune cells, thereby hindering downstream flow cytometric analysis of immune cell subsets.

### Statistical analysis

GraphPad Prism 10.0.3. (GraphPad software Inc.) was used for statistical outlier removal (ROUT, 1%) of all datasets and for the creation of all graphs.

All Univariate Analysis of Variances (ANOVA) (SEX x DIET x AS or SEX x DIET for Fig. S3) were performed with IBM SPSS Statistics followed by a Bonferroni’s adjusted multiple comparisons test. This allowed us to focus on the interaction of sex, diet and adiposity stages. We focused on interactions, as we were not directly interested in sex baseline differences, but mainly on how sex affects diet response (Table 1).

**Table 1:** Overview of sex X diet interaction

	*df*	*F*	*p*						
%vAT/BW	1.53	0.369	0.546						
%scAT/BW	1.53	0.615	0.436						

Cell count	vAT			scAT			Spleen (%)		
	*df*	*F*	*p*	*df*	*F*	*p*	*df*	*F*	*p*
CD19+	1.48	0.925	0.341	1.46	2.878	0.97	1.52	0.706	0.405
CD3+CD4+	1.49	0.289	0.593	1.49	2.835	0.99	1.52	0.127	0.723
CD3+CD8+	1.48	0.390	0.535	1.47	0.849	0.362	1.52	0.012	0.914
Th1 (CXCR3+CCR6−)	1.48	0.016	0.899	1.49	0.586	0.448	1.52	0.05	0.824
Th17 (CCR6+CXCR3−)	1.49	0.210	0.885	1.45	3.616	0.640	1.52	0.053	0.820
Th1/17 (CCR6+CXCR3+)	1.48	0.214	0.645	1.49	0.115	0.736	1.52	0.303	0.584
Treg (CD25+)	1.47	0.711	0.403	1.49	1.856	0.179	1.52	0.135	0.715
CD11b+	1.49	0.392	0.543	1.50	7.032	**0.011**	1.52	0.316	0.576

ASC specking (%)	vAT			scAT			Spleen		
	*df*	*F*	*p*	*df*	*F*	*p*	*df*	*F*	*p*
CD19+	1.48	2.627	0.112	1.43	0.135	0.715	1.51	0.911	0.344
CD3+CD4+	1.49	1.346	0.252	1.46	0.831	0.367	1.50	0.820	0.370
CD3+CD8+	1.48	2.971	0.091	1.46	0.168	0.684	1.51	0.027	0.870
CD11b+	1.49	24.440	** < 0.001**	1.46	3.711	0.060	1.51	0.829	0.367
z-score inflammatory cytokines in blood plasma	*df*	*F*	*p*						
	1.51	1.959	0.168						

Sex X diet interaction are shown from all ANOVA (SEX x DIET X AS), as presented in Supplementary File 1

Significant p-values are in bold

For plasma cytokines, Z-scores of cytokines were obtained with SPSS and an inflammation score was computed in SPSS by averaging the z-score of IL-23, IL-1α, IFN-γ, TNF-α, MCP-1, IL-1β, IL-17A and GM-CSF.

GraphPad Prism was used for the creation of repeated measures mixed-effects model and Bonferroni’s adjusted multiple comparison test for bodyweight over time. Data is expressed as mean ± standard error of the mean (SEM). Differences were considered statistically significant at p ≤ 0.05*, p ≤ 0.005**, p ≤ 0.001***, p ≤ 0.0001****. For clarity, all ANOVA and multiple comparisons tables can be found in Supplementary File 1.

## Results

### Female adiposity is 8 weeks delayed

Literature shows that weight gain in female mice occurs later than in male mice, therefore, this was taken into account for our study design (Fig. 1A) [24–26]. We confirmed that body weight in HFD female mice only statistically increased after 9 weeks of HFD, while male mice show an increase after 1 week (Fig. 1B). To control for these differences in adiposity, male mice were sacrificed at the age of 10, 13, and 18 weeks. The start of sacrificing female mice started 8 weeks after the male mice: 18, 23, and 28 weeks (Fig. 1A, B). At these selected time points, we found that adiposity, defined as weight of the fat depot normalized to total body weight on the day of sacrifice, was not statistically different between male and female mice (Fig. 1C). Interaction analysis indeed revealed that sex did not affect vAT or scAT percentage, or the effect of diet or time (Supplementary File 1). Thus, these time points represent similar adiposity stages (AS) between male (10 weeks: AS1, 13 weeks: AS2 and 18 weeks: AS3) and female (18 weeks: AS1, 23 weeks: AS2, and 28 weeks: AS3) mice.

Taken together, our experimental setup allows us to compare male and female mice based on their adiposity, rather than the time of diet, and to leave the AS variable out of our statistical analyses (sex X diet interactions are summarized in Table 1).

### The number of adaptive immune cells in AT depends on sex and the AS

**Fig. 2:**
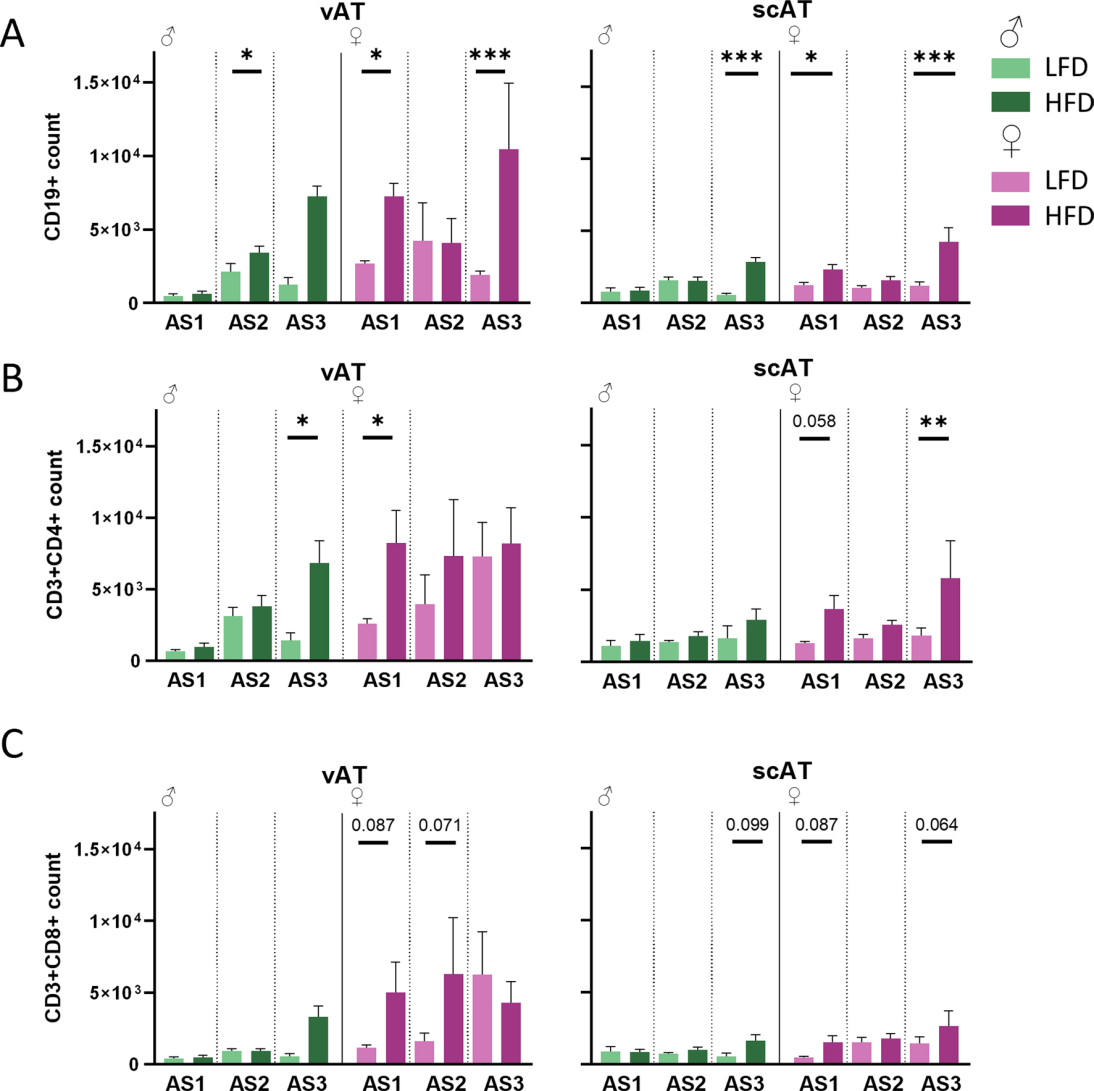
Sex affects the main adaptive immune cell regulation in vAT and scAT differently. **A** CD19^+^ B cells increase in the vAT and scAT of male and female mice with HFD, but the effect is more pronounced in the scAT. **B** CD3^+^CD4^+^ Th cells increase due to DIO in male mice with AS in vAT, but not in scAT. In female mice the number increases in the LFD group in vAT and diminishes the difference due to the diet. In the scAT only at AS3 CD3^+^CD4^+^ cells are upregulated in female mice. **C** CD3^+^CD8^+^ T cells are higher in female mice, but only in the vAT trends of an effect of the diet can be seen. (vAT: n = 6/♂ AS3 HFD, ♀ AS1 HFD, LFD; n = 5/♂ AS1 HFD, AS2 HFD, LFD, AS3 LFD, ♀ AS2 HFD, AS2 LFD (CD19, CD4), AS3 HFD, LFD (CD4, CD8); n = 4/♀ AS2 LFD CD8, AS3 LFD CD19; n = 3/♂ AS1 LFD; scAT: n = 6/♀ AS1 HFD; n = 5/♂ AS1 HFD, LFD (CD4, CD8), AS2 HFD, LFD, AS3 HFD, LFD (CD4), ♀ AS1 LFD, AS2 HFD, LFD, AS3 HFD, LFD; n = 4/♀ AS1 LFD CD19, AS3 LFD CD19, CD8;); p ≤ 0.05*, p ≤ 0.005**, p ≤ 0.001***

First, we looked at changes in the adaptive immune system in response to DIO (Fig. 2). In vAT, frequencies of CD19^+^ and CD4^+^ cells were increased after HFD independently of sex. In scAT, the number of immune cells was generally lower, but sex also impacted how immune cell populations changed over time.

When performing quantification of the different lymphocyte compartments in response to HFD, we identified that in the vAT, the count of CD19^+^ cells increases with adiposity in HFD independent of sex, but it happens at an earlier AS in female mice. In the scAT we see a similar pattern (Fig. 2A). In the vAT, we detected an increased number of Th cells (CD3^+^CD4^+^) in female mice at AS1 and in male mice at AS3 due to the HFD. In the scAT, lower numbers of CD4^+^ T cells were detected, but we did identify an accumulation of CD4^+^ T cells in female mice on HFD (Fig. 2B). In the CD8^+^ T cell compartment (Fig. 2C), we identified a high inter-subject variation of CD8^+^ T cells in the vAT after HFD in female mice. Due to this, no significant differences were identified. In the scAT, we could detect an effect of diet, but looking at both sexes separately, we did not detect a change in CD8^+^ numbers.

**Fig. 3:**
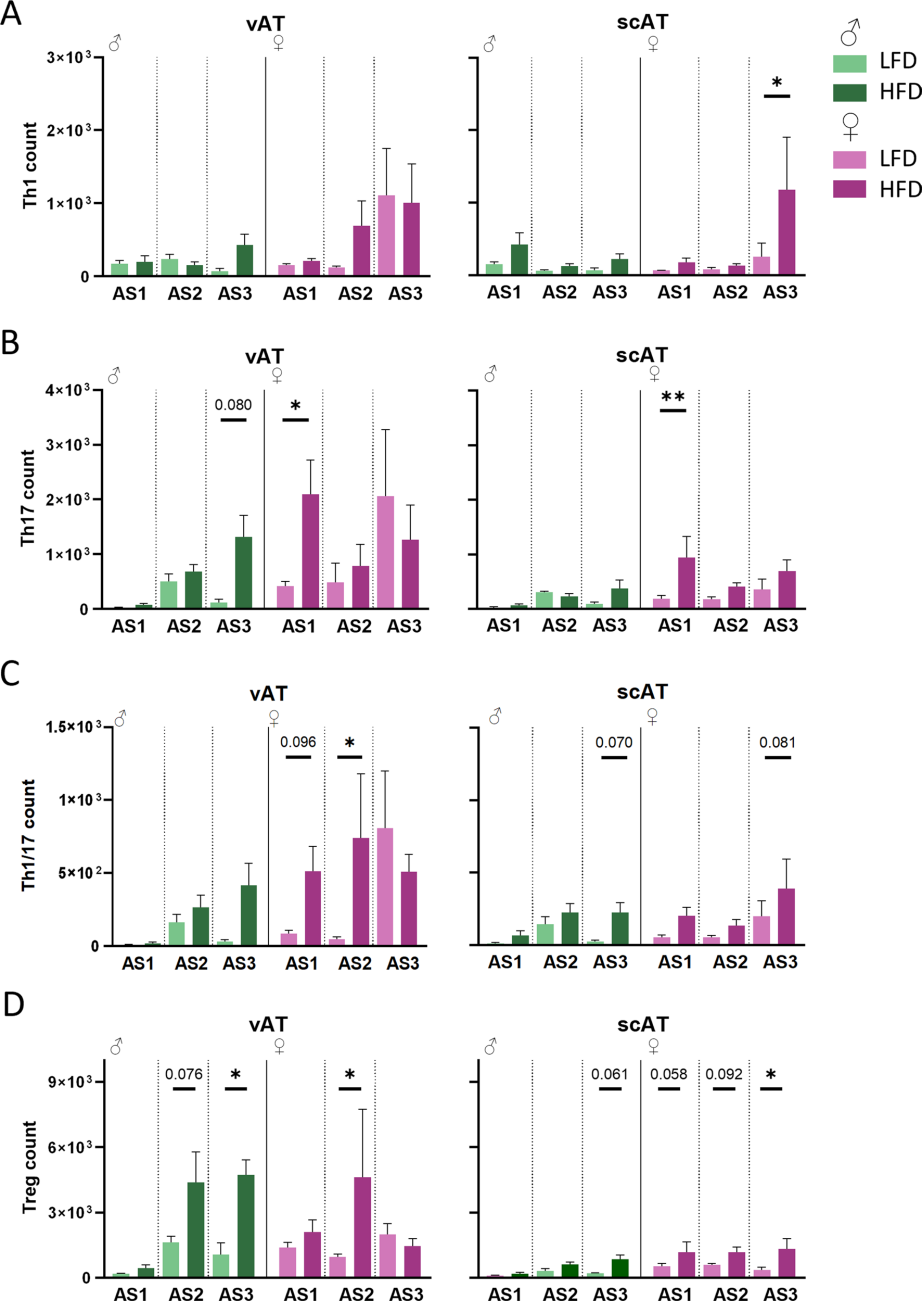
Pro-inflammatory Th cell subsets are differently regulated in male and female mice in AT. **A** Pro-inflammatory Th1 (CXCR3^+^CCR6^−^) **B** Th17 (CCR6^+^CXCR3^−^) and **C** Th17/1 (CXCR3^+^CCR6^+^) cell counts in visceral and subuctaneous fat depots of male and female mice. **D** Treg (CD25^+^) counts in the AT depots. (vAT: n = 6/♂ AS3 HFD, ♀ AS1 HFD (Th17, TH17/1, Treg), LFD; n = 5/♂ AS1 HFD, AS2 HFD, LFD, AS3 LFD, ♀ AS2 HFD, AS2 LFD (Th17), AS3 HFD, LFD); n = 4/♀ AS2 LFD Th1; n = 3/♂ AS1 LFD; scAT: all groups n = 5, only ♀ AS1 HFD n = 6); p ≤ 0.05*, p ≤ 0.005**, p ≤ 0.001***

Within the different Th cell subsets (Fig. 3), DIO did not affect Th1 (CXCR3^+^CCR6^−^) cell numbers in vAT. However, in scAT a clear increase of Th1 cells at AS3 in female mice on HFD was found (Fig. 3A). Importantly, the overall effect of HFD on Th1 levels appeared independent of sex.

As seen in Fig. 3B, changes in Th17 (CCR6^+^CXCR3^−^) cells due to the diet differed between male and female mice based on AS (Fig. 3B). In female mice Th17 are more present at AS1 in vAT and in male mice only a trend of upregulation due to HFD at AS3 was seen. Furthermore, the higher number of Th17 cells at AS1 in HFD female mice in vAT and scAT subsides with a higher AS. In the scAT, a similar trend was found for the effect of sex on the diet response.

For Th1/17 cells (CCR6^+^CXCR3^+^), we identified that sex affects how mice react to DIO in different AS stages in vAT. The number of Th1/17 cells at AS1 was very low in male mice, but with increasing adiposity, they tended to accumulate in the HFD group (Fig. 3C). In females, Th1/17 accumulated at and early AS and were significantly elevated at AS2. However, this difference subsided in AS3, due to an increase in LFD mice. In the scAT the amount of these cells was very low, although they tended to accumulate in this depot over time. While multiple comparisons did not identify a significant difference between the dietary interventions, a simple effect of diet was observed when combining the data of male and female mice from all AS. Overall, in female mice, the upregulation of pro-inflammatory Th cell subsets was diminished with increasing AS. In the scAT, an upregulation was only identified in female HFD mice.

Regulatory T cells (Tregs, CD25^+^) did not show an interaction with sex, even though especially in male mice clear changes in Treg numbers were observed due to HFD (Fig. 3D). Pairwise comparisons reveal that at AS2 Tregs were increased in female mice on HFD. In male mice this occurred at AS3, while in female mice Tregs subsided to baseline level. In scAT we found a strong overall effect of diet. At all adiposity stages a trend toward a higher Treg count in the HFD fed female mice was observed, which was significant at AS3. In the male mice, only at AS3, Tregs tended to be increased by DIO.

Taken together, CD19^+^ cells were increased in the DIO model of both sexes, but at an earlier AS in female mice. In the T cell compartments, the number of cells was also increased at AS1 and AS2 in female mice, while at AS3 no differences were observed due to an increase in T cells in control-diet fed animals. In contrast, male mice displayed a steady increase. In the scAT only CD4^+^ T cells displayed changes between different AS. To reveal whether the observed changes in the immune compartments were specific to AT in these mice, we also analysed the same cell populations in the spleens (Fig. S2). Overall, no clear skewing to pro-inflammatory cells due to HFD was detected. Furthermore, the identified differences were not in line with the changes in the fat depots, showing that the described changes in the white fat depots were not due to general changes in a primary lymphoid organ, and therefore occur independently.

### HFD upregulates myeloid cells in different fat depots based on sex

**Fig. 4:**
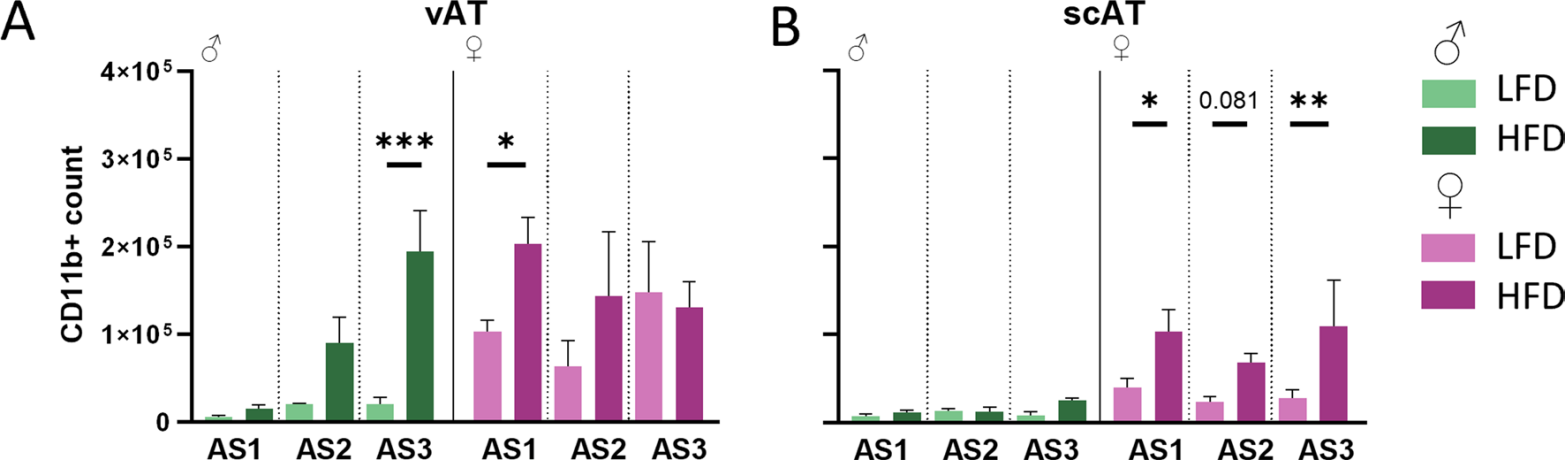
Myeloid cells (CD11b^+^) are upregulated in different AT depots depending on sex. **A** CD11b^+^ cells are increasing with AS in male mice, while they are decreasing in female mice. **B** In male mice CD11b^+^ cells are not affected by DIO. But in female mice DIO upregulates them at all AS. (n = 6/♂ AS1 HFD scAT, AS3 HFD vAT, ♀ AS1 HFD, LFD vAT; n = 5/♂ AS1 HFD vAT, AS2 HFD, LFD scAT, AS3 HFD scAT, LFD vAT ♀ AS1 LFD scAT, AS2 HFD, LFD, AS3 HFD, LFD; n = 4/♂ AS2 LFD vAT, AS3 LFD scAT; n = 3/♂ AS1 LFD vAT); p ≤ 0.05*, p ≤ 0.005**, p ≤ 0.001***

The main immune cell populations in the AT are myeloid cells (CD11b^+^) [7, 27], therefore we assessed their overall presence in the AT depots (Fig. 4). Interestingly, sex strongly affected how diet changed this cell compartment at different AS in vAT. Myeloid cells were increased with adiposity in male mice, while they remained stable in the LFD group. In female mice, myeloid cell numbers were higher in the HFD group at AS1, but with increased adiposity these levels decreased and the numbers in the LFD group increased. In contrast, in scAT, the amount of CD11b^+^ cells was unaffected in male mice, while HFD lead to a stronger upregulation in female mice, reflecting a clear effect of sex on the diet response (SEX x DIET: F(2,50) = 7.03, p = 0.011; Table 1). In short, in male mice dietary interventions impact myeloid cells in the vAT, but in females this effect is pronounced in the scAT.

### Sex differences in AT depots are independent of time on diet and age

Interestingly, AS3 in male mice and AS1 in female mice had similar patterns of immune cell accumulation in the AT depots. Since these groups differ in AS but reflected the same diet duration and age (Fig. 1), we compared these groups directly to each other (Fig. S3). Although some immune cell populations are not significantly different between male and female mice after 10 weeks, myeloid cells and Tregs in the vAT were only increased in male mice. This comparison shows that even at the same age and after the same dietary intervention period, some sex differences are apparent.

### Only in male mice, HFD leads to inflammasome activation in myeloid cells of vAT

**Fig. 5:**
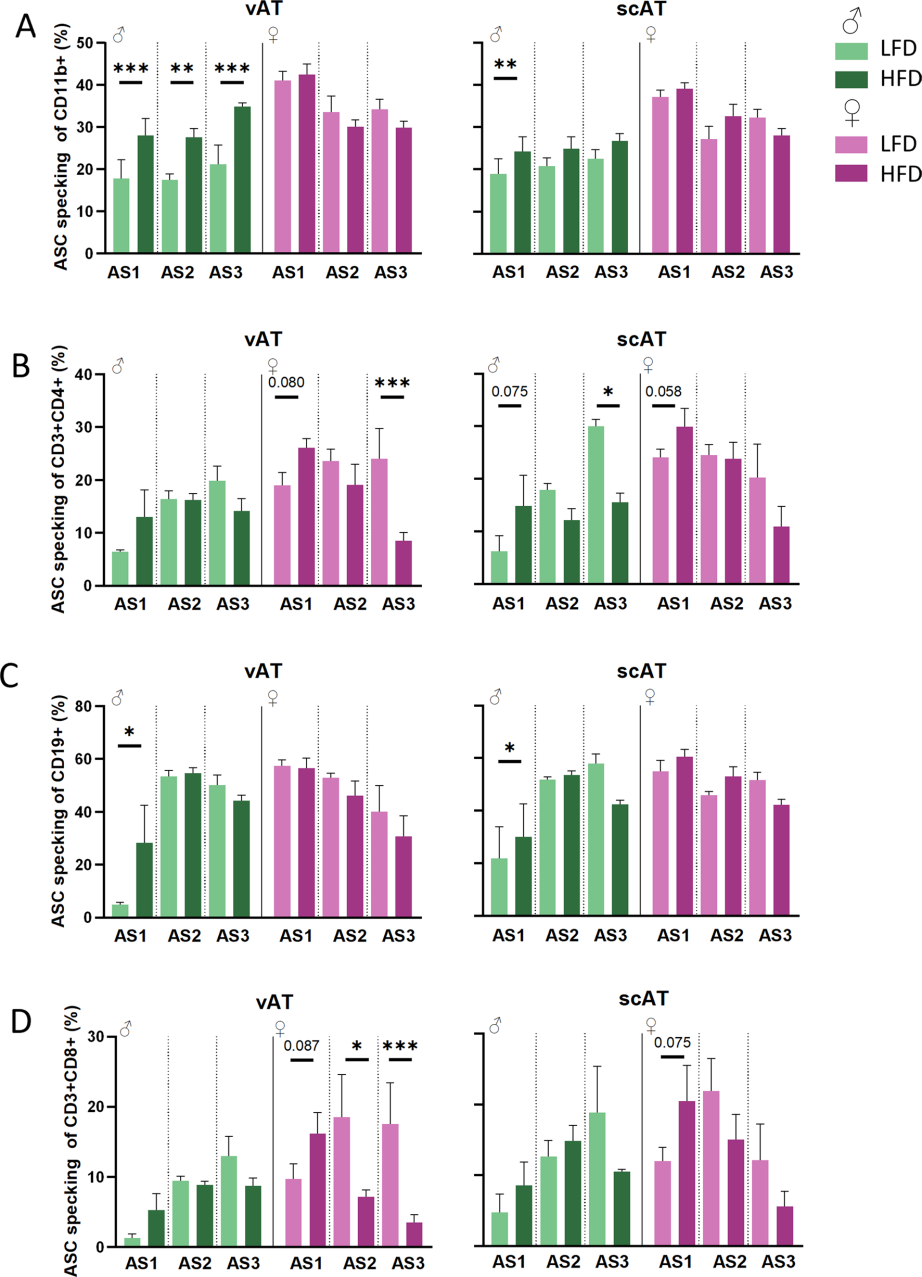
Only in male mice, CD11b^+^ cells inflammasome activation is upregulated with DIO. **A** Myeloid cells (CD11b^+^) have an upregulated inflammasome activation due to DIO in male mice. In Th cells (**B**, CD3^+^CD4^+^), B cells (**C**, CD19^+^) and CD8^+^ T cells (**D**) changes in inflammasome activation are not clearly linked to adiposity. (n = 3–6/group, see Figs. 2 + 4); p ≤ 0.05*, p ≤ 0.005**, p ≤ 0.001***

Next, we investigated inflammasome activation in the different cell compartments. In male mice, DIO led to inflammasome activation in vAT myeloid cells. On the contrary, diet had no effect on inflammasome activation in female mice. However, the baseline % of cells with an activated inflammasome was always higher in female mice if compared to male mice. In addition, sex strongly affected inflammasome activation due to diet and AS. In the scAT the differences were smaller, but overall, a similar trend can be seen (Fig. 5A).

In cells of the adaptive immune system of male and female mice, we found a trend towards higher inflammasome activation at the lowest AS in both sexes on HFD, but at higher adiposity, no clear inflammasome activation in AT could be identified (Fig. 5B–D). Similar findings were observed in the spleens of these mice (Fig. S4). Together, this suggests that inflammasome activation is male-specific in DIO and most prominent in the vAT.

### Systemic inflammatory cytokines mimic inflammation in the vAT

**Fig. 6:**
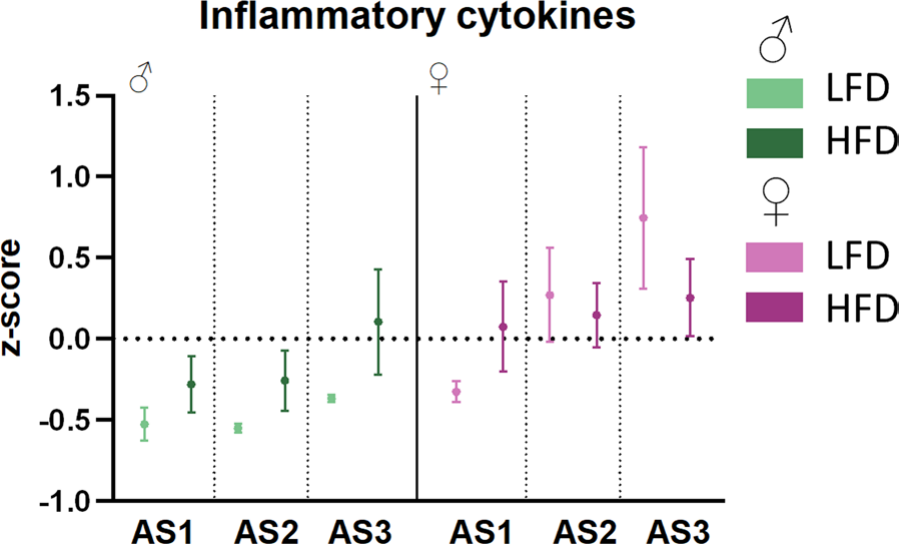
Pro-inflammatory cytokines in the blood plasma mirror the vAT inflammatory stage. Based on a z-score of pro-inflammatory cytokines (IL-23, IL-1α, IFN-γ, TNF- α, MCP-1, IL-1β, IL-17A, GM-CSF), no significant change in systemic inflammation due to adiposity can be found. (n = 6/♂ AS1 HFD, AS3 HFD ♀ AS1 HFD, LFD, AS3 LFD; n = 5/♂ AS1 LFD, AS2 HFD,♀ AS2 HFD, LFD, AS3 HFD; n = 4 ♂ AS2 LFD, AS3 LFD)

To understand if the differences in the AT depots correlated with systemic inflammatory changes, we assessed pro-inflammatory cytokines in the blood plasma. Since HFD leads to low grade chronic inflammation, with an upregulation of several different cytokines, we computed an inflammation z-score (including IL-23, IL-1α, IFN-γ, TNF, MCP-1, IL-1β, GM-CSF, IL-17A) to get an overview of the inflammatory state (Fig. 6). Only simple effects of the different ASs could be observed, but no effect of the dietary intervention or an effect of sex on DIO. There is a trend that the z-score increases for male mice on HFD depending on AS. In contrast, the same seems to be the case for female mice on LFD. However, from the chosen cytokines, not all were detectable in the blood plasma of each mouse, leading to a high variation in different groups.

Given its direct correlation with inflammasome activation, we additionally assessed IL-1β levels separately from the compiled z-score. In line with the ASC specking data (Fig. 5), HFD increases IL-1β levels in plasma of male mice, although this increase did not reach statistical significance (Fig. S5). In female mice, however, there was an increase in IL-1β plasma levels due to HFD at AS1 and AS2, which is in contrast to the overall finding of ASC-specking in AT-derived immune cells. At AS3, no effect of diet could be observed in female mice, which is in line with ASC-specking.

## Discussion

The objective of this study was to uncover how sex affects immune cell populations in the AT during different adiposity stages (ASs). As it is widely known that young male mice on HFD gain more adipose tissue in a shorter period than female mice, we show that by taking this delay into account, adiposity is comparable and that HFD also leads to a stable weight gain in female mice. Therefore, we propose that this is no valid reason to directly exclude female obesity models from preclinical research.

However, we did identify differences in the immune cell populations in the vAT and scAT, especially in how they change depending on the AS. The overall changes in pro-inflammatory cells in the vAT were in accordance with the pro-inflammatory cytokine profile in the blood plasma. Looking at the major immune cells in vAT, we saw that in male mice obesity increases the number of all pro-inflammatory cell populations, while in female mice already at AS1, a more pronounced effect was seen, but with the exception of CD19^+^ cells, these changes were temporary as there was a consistent reverse trend at AS3. Furthermore, in female mice, the number of cells in the LFD groups was less stable. It could be argued, that part of this is due to fluctuations of the estrous cycle as estrogens are considered to play a pronounced role in immunoregulation in obesity [28, 29]. However, in the blood circulation, it was previously shown that the reproductive cycle does not affect immune cell populations and that the changes of plasma cytokines cannot be explained by this [30, 31]. Therefore, these changes may be due to other unidentified factors. One factor could be resistin-like molecule α (RELMα), as RELMα deficiency was shown to lead to a higher weight gain accompanied by more pro-inflammatory macrophages in the vAT of female mice [32]. In another study, monocyte trafficking in vAT in female mice on a 12 to 16-week HFD was decreased compared to male mice in the same conditions [29]. While these studies improve our knowledge of sex dimorphism in obesity models, they still focus on vAT and information on scAT sex differences is scarce.

We saw that the immune cell numbers in the AT were similar between early obesity in female mice (AS1) and ongoing obesity in male mice (AS3). This would indicate that an increase in immune cells in the vAT depot is not depending on fat depot weight (female HFD AS1: 580 ± 254 (SD) mg, male HFD AS3: 2085 ± 395 (SD) mg), but rather on the period of the dietary intervention or the age of the mice. As we looked at the effect of adiposity, an effect of age cannot be eliminated, since female mice were up to 10 weeks older at the time of their sacrifice. Moreover, the duration of the diet may also affect immune populations [33, 34]. Most studies investigating effects of age on the immune system include an age difference larger than 6 months. Therefore, our findings cannot be easily compared to previous studies on aging. Thus, further research into the effect of short-term aging on AT inflammation in male and female mice is necessary.

Interestingly, DIO induced inflammation specifically in scAT of female mice, while the effect of diet in this depot was minimal for male mice. For CD11b^+^ myeloid cells, this effect is even more pronounced than in vAT. Due to the set-up of the experiment, we cannot exactly determine which myeloid cells are affected by the DIO and future research should help understand sex effects on the innate immune system. Although there is a general consent that vAT is the most inflammatory fat depot, a review by Delany and Santosa (2022) highlights that in premenopausal women obesity leads to more changes in the scAT than in men [35]. Furthermore, Delany and Santosa concluded that in women scAT expansion may be linked to a higher type 2 diabetes mellitus risk rather than vAT expansion. Our results contribute to these findings and suggest that increased inflammation specifically in scAT of women may contribute to these observations. This is of major interest, as it is known that there is a different fat depot composition based on sex in humans. Previous research has shown that males preferentially store energy in vAT, while females accumulate adipose tissue primarily in the subcutaneous depot, which pronounces the immune infiltration seen in our study [36–38]. In addition, there is evidence that sex hormones are not the only explanation, as the exclusion of gonadal hormones in mice still leads to differences based on X chromosome dosage [39]. In our DIO model, we also see that sex may affect which fat depot is mainly affected by adipose tissue inflammation.

Our mouse model allowed us to assess inflammasome activation. Strikingly, inflammasome activation in the myeloid cell compartment was upregulated by HFD only in vAT, and to a lesser extent in scAT, of male mice at each AS. This correlated with IL-1β plasma levels in male, but not female mice. In female mice, an increase in AS did not correlate with an increase in ASC specking (i.e. inflammasome activation). Moreover, enhanced inflammasome activation was not observed in splenocytes or in female mice. Therefore, activation of the inflammasome seems sex and AT-specific. Previous studies already showed evidence of increased NLRP3 inflammasome activation in mice in relation to obesity [15]. However, these conclusions were drawn from mRNA expression or IL-1β excretion, and only male mice were investigated. In our study, by using ASC-reporter mice, we were able to discriminate between total expression of ASC and ASC specking (i.e. inflammasome activation). We now contribute to these findings by showing that inflammasome activation is confined to myeloid cells. Publications investigating diet-induced inflammasome activation in female mice are scarce. One published study looked at the effect of obesity on inflammasome activation in female mice using ovariectomized mice [40]. There, ASC gene expression was increased due to HFD. Another study in 12-month-old female mice saw that *Nlrp3* mRNA expression decreased with weight loss and correlated with body weight [10]. To gain deeper insight into the sex differences of inflammasome activation in a DIO model, our understanding of the effect of sex on physiological function of the NLRP3 inflammasome needs to be better characterized. Future studies should investigate whether the lack of inflammasome activation is a reason why female mice are protected against severe systemic and metabolic comorbidities, as previous studies linked NLRP3 activation with insulin resistance and atherosclerosis [10, 15, 16, 19].

Lastly, we looked at pro-inflammatory cytokines in the circulation with a combined z-score of inflammatory cytokines. We opted to only include cytokines that are described as exclusively pro-inflammatory in literature. Interestingly, the systemic inflammation score is in line with the amount of pro-inflammatory CD3^+^ and myeloid cells in the vAT at the different adiposity stages, showing an upregulation due to DIO in male mice. Therefore, vAT inflammation seems to be closer linked to systemic inflammation, as the CD11b^+^ accumulation in scAT of female HFD mice did not relate to the z-score. However, in LFD-fed male mice, there is almost no variation in the cytokine profile, and over time there is an increase in the z-score for HFD. To our surprise, there is a trend of an increase in low-grade inflammation in female mice on LFD, while the z-score in the HFD groups is quite stable between AS. Further research into unravelling the role of different cells in different fat depots and their release of cytokines at different adiposity stages is needed to comprehend this complex interplay.

Taken together, we show that adiposity affects pro-inflammatory cells earlier in female mice, but these changes were diminished with increased adiposity in vAT. Furthermore, immune cell accumulation in the scAT was mainly found in female HFD mice. For the first time, we could show that inflammasome activation is restricted to the myeloid cells in male mice. Pro-inflammatory cytokine levels in the plasma mirror the inflammatory state of the vAT, and changes in the spleen, a primary lymphoid organ, are not correlated with the ongoing changes in the AT depots.

Based on our findings, we suggest that the timing of DIO should be thoroughly considered for planned studies and we propose that the effect of sex on different fat depots in health and disease should be further investigated. We found changes due to DIO in scAT of female mice, but most studies focus on vAT and the effect of changes in scAT are less well understood. A major strength of this study is the use of adiposity stage instead of time on diet as “time points”, on the other hand, the low sample size is a limitation and further investigations should be initiated to understand this complex effect of sex and diet intervention on the immune system. As mentioned before, this model disregards the effect of age and time on diet, although we have shown that even when these factors are taken into account, there is sexual dimorphism in the AT inflammation. Therefore, this set-up is perfectly suited to assess inflammation at a similar adiposity between male and female mice. Understanding the limitations of the model used, we have provided important insights into the latter, which holds great implications for future studies into obesity using preclinical models. Gaining more insights into sex differences could help us to determine why adiposity in men and women increases the risk for different diseases in distinct ways. Further exploration could lead to finding more specific therapy targets for patients with metabolic syndrome or obesity.

## Supplementary Information

Supplementary Material 1.

Supplementary Material 2. Table S1. Diet compositions.

Supplementary Material 3. Figure S1. Flow cytometry gating used for the whole experimental setup. Of all recorded cells a FSC-A subset corresponding to the Moxi^TM^ counted cell number was used for singlet and live/dead gating. Lymphocytes (based on size) and myeloid cells (based on CD11b^+^) were gated from all CD45^+^ cells. CD19^+^ and CD3^+^ were used to determine lymphocyte lineage. To further gate T cells, CD25^+^ cells were excluded from the CD4^+^/CD8^+^ gating. The Th cell compartment was further divided into Th1 (CXCR3^+^CCR6^-^), Th17 (CCR6^+^CXCR3^-^) and Th1/17 (CXCR3^+^CCR6^+^). Of the major cell populations inflammasome activation was gated based on ASC specking.

Supplementary Material 4.

Supplementary Material 5. Figure S3: Sex dimorphism is apparent at similar time on diet and age. Accumulation of B cells **(A)**, CD3^+^CD4^+^ Th cells **(B)**, CD3^+^CD8^+^ T cells **(C)**, CD11b^+^ myeloid cells **(D)**, Th1 **(E)**, Th17 **(F)**, Th1/17 **(G)** and CD25^+^ Treg **(H)** in vAT and scAT. Differences between male and female mice are seen in the scAT for CD11b^+^ myeloid cells and CD25^+^ Treg cells in vAT. (n=4-6/group, see Figure 2, 3, 4); p≤0.05*/#, p≤0.005**/##, p≤0.001***/###; *=changes based on diet; #=changes based on sex.

Supplementary Material 6. Figure S4: In the spleen inflammasome activation in immune cells is not affected by the dietary intervention. % of ASC specking cells of CD11b^+^ myeloid cells **(A)**, CD3^+^CD4^+^ Th cells **(B)**, CD19^+^ B cells **(C)** and CD3^+^CD8^+^ T cells **(D)** of harvested splenocytes. In comparison to vAT and scAT, ASC specking due to inflammasome activation is lower in all immune cell subsets. (n=5-6/group, see Figure S2).

Supplementary Material 7. Figure S5: IL-1β levels in blood plasma correlate with inflammasome activation in AT-derived immune cells of male mice, not in female mice. Concentration of IL-1β plasma levels at different AS in male and female mice after LFD or HFD. (n=6/♂ AS1 HFD, AS3 HFD ♀ AS1 HFD, LFD, AS3 LFD; n=5/♂ AS1 LFD, AS2 HFD, LFD, ♀ AS2 HFD, LFD, AS3 HFD; n=4/♂ AS3 LFD, ).

## Data Availability

Statistical analyses are provided in the supplementary materials. Raw data (including.fcs files) will be shared upon reasonable request.

## Abbreviations

AS: Adiposity stage
ASC: Apoptosis-associated speck-like protein containing a C-terminal caspase recruitment domain
AT: Adipose tissue
BMI: Body mass index
DIO: Diet induced obesity
FBS: Fetal bovine serum
HFD: High fat diet
IFN: Interferon
IL: Interleukin
LFD: Low fat diet
NLRP3: Nucleotide-binding domain, leucine-rich-containing family, pyrin domain-containing-3
scAT: Subcutaneous adipose tissue
SVF: Stromal vascular fraction
Th: T helper
TNF: Tumor necrosis factor
Treg: Regulatory T cell
vAT: Visceral adipose tissue
WHO: World Health Organization
