# Supplementary material for: Sex differentially affects pro-inflammatory cell subsets in adipose tissue depots in a diet induced obesity model
Source: Biol Sex Differ. 2024 Dec 18;15:105. doi: 10.1186/s13293-024-00677-1 (PMC11657622; doi:10.1186/s13293-024-00677-1)
Supplement: Supplementary file 1 — Supplementary Material 1. [file 13293_2024_677_MOESM1_ESM.pdf]

% vAT/BW

Tests of Between-Subjects Effects

Dependent Variable: vAT_perc

Source	Type III Sum of Squares	df	Mean Square	F	Sig.
Corrected Model	115,309[a]	11	10,483	19,081	<,001
Intercept	451,305	1	451,305	821,465	<,001
SEX	,015	1	,015	,028	,868
DIET	64,603	1	64,603	117,590	<,001
AS	30,989	2	15,495	28,203	<,001
SEX * DIET	,203	1	,203	,369	,546
SEX * AS	,162	2	,081	,148	,863
DIET * AS	19,833	2	9,917	18,050	<,001
SEX * DIET * AS	,202	2	,101	,184	,833
Error	29,118	53	,549		
Total	603,862	65			
Corrected Total	144,427	64			

a. R Squared = ,798 (Adjusted R Squared = ,757)

Pairwise Comparisons

Dependent Variable: vAT_perc

SEX	AS	(I) DIET	(J) DIET	Mean Difference (I-J)	Std. Error	Sig.[b]	95% Confidence Interval for Difference[b]	
							Lower Bound	Upper Bound
female	AS1	LFD	HFD	-,500	,428	,248	-1,358	,358
		HFD	LFD	,500	,428	,248	-,358	1,358
	AS2	LFD	HFD	-1,950*	,469	<,001	-2,890	-1,010
		HFD	LFD	1,950*	,469	<,001	1,010	2,890
	AS3	LFD	HFD	-3,219*	,449	<,001	-4,120	-2,319
		HFD	LFD	3,219*	,449	<,001	2,319	4,120
male	AS1	LFD	HFD	-,938*	,449	,041	-1,838	-,038
		HFD	LFD	,938*	,449	,041	,038	1,838
	AS2	LFD	HFD	-1,860*	,469	<,001	-2,800	-,920
		HFD	LFD	1,860*	,469	<,001	,920	2,800
	AS3	LFD	HFD	-3,544*	,449	<,001	-4,445	-2,644
		HFD	LFD	3,544*	,449	<,001	2,644	4,445

Based on estimated marginal means

*. The mean difference is significant at the 0,05 level.

b. Adjustment for multiple comparisons: Bonferroni.

% scAT/BW

Tests of Between-Subjects Effects

Dependent Variable: scAT_perc

Source	Type III Sum of Squares	df	Mean Square	F	Sig.
Corrected Model	44,296[a]	11	4,027	17,095	<,001
Intercept	179,182	1	179,182	760,671	<,001
SEX	,443	1	,443	1,879	,176
DIET	22,565	1	22,565	95,795	<,001
AS	14,322	2	7,161	30,401	<,001
SEX * DIET	,145	1	,145	,615	,436
SEX * AS	,161	2	,081	,342	,712
DIET * AS	7,236	2	3,618	15,359	<,001
SEX * DIET * AS	,248	2	,124	,527	,594
Error	12,485	53	,236		
Total	239,297	65			
Corrected Total	56,780	64			

a. R Squared = ,780 (Adjusted R Squared = ,734)

Pairwise Comparisons

Dependent Variable: scAT_perc

SEX	AS	(I) DIET	(J) DIET	Mean Difference (I-J)	Std. Error	Sig.[b]	95% Confidence Interval for Difference[b] Lower Bound	Upper Bound
female	AS1	LFD	HFD	-,478	,280	,094	-1,040	,084
		HFD	LFD	,478	,280	,094	-,084	1,040
	AS2	LFD	HFD	-1,020*	,307	,002	-1,636	-,404
		HFD	LFD	1,020*	,307	,002	,404	1,636
	AS3	LFD	HFD	-2,336*	,294	<,001	-2,925	-1,746
		HFD	LFD	2,336*	,294	<,001	1,746	2,925
male	AS1	LFD	HFD	-,582	,294	,053	-1,171	,007
		HFD	LFD	,582	,294	,053	-,007	1,171
	AS2	LFD	HFD	-,840*	,307	,008	-1,456	-,224
		HFD	LFD	,840*	,307	,008	,224	1,456
	AS3	LFD	HFD	-1,843*	,294	<,001	-2,432	-1,254
		HFD	LFD	1,843*	,294	<,001	1,254	2,432

Based on estimated marginal means

*. The mean difference is significant at the 0,05 level.

b. Adjustment for multiple comparisons: Bonferroni.

Immune cell amount in adipose tissue depots

CD19+

vAT

Tests of Between-Subjects Effects

Dependent Variable: vAT_CD19

Source	Type III Sum of Squares	df	Mean Square	F	Sig.
Corrected Model	513001106,1[a]	11	46636464,189	3,499	,001
Intercept	845485258,77	1	845485258,77	63,432	<,001
SEX	95922227,235	1	95922227,235	7,196	,010
DIET	167854152,50	1	167854152,50	12,593	<,001
AS	61206418,207	2	30603209,103	2,296	,112
SEX * DIET	12333136,249	1	12333136,249	,925	,341
SEX * AS	24466999,379	2	12233499,690	,918	,406
DIET * AS	117534215,26	2	58767107,632	4,409	,017
SEX * DIET * AS	22033551,678	2	11016775,839	,827	,444
Error	639794607,58	48	13329054,325		
Total	2140242921,5	60			
Corrected Total	1152795713,7	59			

a. R Squared = ,445 (Adjusted R Squared = ,318)

Pairwise Comparisons

Dependent Variable: vAT_CD19

SEX	AS	(I) DIET	(J) DIET	Mean Difference (I-J)	Std. Error	Sig.[b]	95% Confidence Interval for Difference[b]	
							Lower Bound	Upper Bound
female	AS1	LFD	HFD	-4583,500*	2107,847	,035	-8821,610	-345,390
		HFD	LFD	4583,500*	2107,847	,035	345,390	8821,610
	AS2	LFD	HFD	150,240	2309,030	,948	-4492,377	4792,857
		HFD	LFD	-150,240	2309,030	,948	-4792,857	4492,377
	AS3	LFD	HFD	-8542,139*	2449,097	,001	-13466,378	-3617,901
		HFD	LFD	8542,139*	2449,097	,001	3617,901	13466,378
male	AS1	LFD	HFD	-151,908	2666,239	,955	-5512,740	5208,924
		HFD	LFD	151,908	2666,239	,955	-5208,924	5512,740
	AS2	LFD	HFD	-1314,672	2309,030	,572	-5957,289	3327,945
		HFD	LFD	1314,672	2309,030	,572	-3327,945	5957,289
	AS3	LFD	HFD	-5974,627*	2210,728	,009	-10419,594	-1529,659
		HFD	LFD	5974,627*	2210,728	,009	1529,659	10419,594

Based on estimated marginal means

*. The mean difference is significant at the 0,05 level.

b. Adjustment for multiple comparisons: Bonferroni.

scAT

Tests of Between-Subjects Effects

Dependent Variable: scAT_CD19

Source	Type III Sum of Squares	df	Mean Square	F	Sig.
Corrected Model	58040430,79[a]	11	5276402,799	7,140	<,001
Intercept	152604230,99	1	152604230,99	206,504	<,001
SEX	4771341,034	1	4771341,034	6,457	,014
DIET	19338247,552	1	19338247,552	26,168	<,001
AS	9260591,079	2	4630295,539	6,266	,004
SEX * DIET	2126722,614	1	2126722,614	2,878	,097
SEX * AS	4908198,527	2	2454099,264	3,321	,045
DIET * AS	16115919,321	2	8057959,661	10,904	<,001
SEX * DIET * AS	151203,663	2	75601,832	,102	,903
Error	33993545,720	46	738990,124		
Total	255855218,53	58			
Corrected Total	92033976,512	57			

a. R Squared = ,631 (Adjusted R Squared = ,542)

Pairwise Comparisons

Dependent Variable: scAT_CD19

SEX	AS	(I) DIET	(J) DIET	Mean Difference (I-J)	Std. Error	Sig.[b]	95% Confidence Interval for Difference[b] Lower Bound	Upper Bound
female	AS1	LFD	HFD	-1108,267*	520,541	,039	-2156,062	-60,472
		HFD	LFD	1108,267*	520,541	,039	60,472	2156,062
	AS2	LFD	HFD	-528,400	543,687	,336	-1622,786	565,986
		HFD	LFD	528,400	543,687	,336	-565,986	1622,786
	AS3	LFD	HFD	-3008,200*	543,687	<,001	-4102,586	-1913,814
		HFD	LFD	3008,200*	543,687	<,001	1913,814	4102,586
male	AS1	LFD	HFD	-58,873	576,668	,919	-1219,645	1101,899
		HFD	LFD	58,873	576,668	,919	-1101,899	1219,645
	AS2	LFD	HFD	35,500	576,668	,951	-1125,272	1196,272
		HFD	LFD	-35,500	576,668	,951	-1196,272	1125,272
	AS3	LFD	HFD	-2308,000*	576,668	<,001	-3468,772	-1147,228
		HFD	LFD	2308,000*	576,668	<,001	1147,228	3468,772

Based on estimated marginal means

*. The mean difference is significant at the 0,05 level.

b. Adjustment for multiple comparisons: Bonferroni.

CD3+CD4+

vAT

Tests of Between-Subjects Effects

Dependent Variable: vAT_CD4

Source	Type III Sum of Squares	df	Mean Square	F	Sig.
Corrected Model	450709530,2[a]	11	40973593,652	2,257	,026
Intercept	1214902720,3	1	1214902720,3	66,908	<,001
SEX	178374462,03	1	178374462,03	9,824	,003
DIET	110518122,38	1	110518122,38	6,087	,017
AS	78067612,313	2	39033806,157	2,150	,127
SEX * DIET	5255556,907	1	5255556,907	,289	,593
SEX * AS	14069339,423	2	7034669,712	,387	,681
DIET * AS	3835005,683	2	1917502,842	,106	,900
SEX * DIET * AS	64186882,086	2	32093441,043	1,767	,181
Error	889727358,85	49	18157701,201		
Total	2702416760,0	61			
Corrected Total	1340436889,0	60			

a. R Squared = ,336 (Adjusted R Squared = ,187)

Pairwise Comparisons

Dependent Variable: vAT_CD4

SEX	AS	(I) DIET	(J) DIET	Mean Difference (I-J)	Std. Error	Sig.[b]	95% Confidence Interval for Difference[b] Lower Bound	Upper Bound
female	AS1	LFD	HFD	-5660,600*	2460,197	,026	-10604,550	-716,650
		HFD	LFD	5660,600*	2460,197	,026	716,650	10604,550
	AS2	LFD	HFD	-3384,920	2695,010	,215	-8800,746	2030,906
		HFD	LFD	3384,920	2695,010	,215	-2030,906	8800,746
	AS3	LFD	HFD	-942,036	2695,010	,728	-6357,862	4473,790
		HFD	LFD	942,036	2695,010	,728	-4473,790	6357,862
male	AS1	LFD	HFD	-337,279	3111,930	,914	-6590,937	5916,378
		HFD	LFD	337,279	3111,930	,914	-5916,378	6590,937
	AS2	LFD	HFD	-668,440	2695,010	,805	-6084,266	4747,386
		HFD	LFD	668,440	2695,010	,805	-4747,386	6084,266
	AS3	LFD	HFD	-5405,732*	2580,276	,041	-10590,991	-220,473
		HFD	LFD	5405,732*	2580,276	,041	220,473	10590,991

Based on estimated marginal means

*. The mean difference is significant at the 0,05 level.

b. Adjustment for multiple comparisons: Bonferroni.

scAT

Tests of Between-Subjects Effects

Dependent Variable: scAT_CD4

Source	Type III Sum of Squares	df	Mean Square	F	Sig.
Corrected Model	102113541,0[a]	11	9283049,177	2,252	,026
Intercept	306230498,04	1	306230498,04	74,295	<,001
SEX	18274067,402	1	18274067,402	4,433	,040
DIET	36679868,022	1	36679868,022	8,899	,004
AS	19019775,481	2	9509887,740	2,307	,110
SEX * DIET	11684894,154	1	11684894,154	2,835	,099
SEX * AS	2622859,200	2	1311429,600	,318	,729
DIET * AS	9798179,133	2	4899089,567	1,189	,313
SEX * DIET * AS	3132826,915	2	1566413,457	,380	,686
Error	201969589,54	49	4121828,358		
Total	617456550,27	61			
Corrected Total	304083130,49	60			

a. R Squared = ,336 (Adjusted R Squared = ,187)

Pairwise Comparisons

Dependent Variable: scAT_CD4

SEX	AS	(I) DIET	(J) DIET	Mean Difference (I-J)	Std. Error	Sig.[b]	95% Confidence Interval for Difference[b]	
							Lower Bound	Upper Bound
female	AS1	LFD	HFD	-2383,133	1229,364	,058	-4853,634	87,367
		HFD	LFD	2383,133	1229,364	,058	-87,367	4853,634
	AS2	LFD	HFD	-930,000	1284,029	,472	-3510,354	1650,354
		HFD	LFD	930,000	1284,029	,472	-1650,354	3510,354
	AS3	LFD	HFD	-3974,800*	1284,029	,003	-6555,154	-1394,446
		HFD	LFD	3974,800*	1284,029	,003	1394,446	6555,154
male	AS1	LFD	HFD	-350,803	1284,029	,786	-2931,156	2229,551
		HFD	LFD	350,803	1284,029	,786	-2229,551	2931,156
	AS2	LFD	HFD	-409,000	1284,029	,751	-2989,354	2171,354
		HFD	LFD	409,000	1284,029	,751	-2171,354	2989,354
	AS3	LFD	HFD	-1269,400	1284,029	,328	-3849,754	1310,954
		HFD	LFD	1269,400	1284,029	,328	-1310,954	3849,754

Based on estimated marginal means

*. The mean difference is significant at the 0,05 level.

b. Adjustment for multiple comparisons: Bonferroni.

CD3+CD8+

vAT

Tests of Between-Subjects Effects

Dependent Variable: vAT_CD8

Source	Type III Sum of Squares	df	Mean Square	F	Sig.
Corrected Model	287067524,6[a]	11	26097047,692	1,813	,078
Intercept	394113561,60	1	394113561,60	27,381	<,001
SEX	130209868,96	1	130209868,96	9,046	,004
DIET	35135969,040	1	35135969,040	2,441	,125
AS	33838815,923	2	16919407,962	1,175	,317
SEX * DIET	5611866,719	1	5611866,719	,390	,535
SEX * AS	1190648,305	2	595324,153	,041	,960
DIET * AS	10835988,375	2	5417994,187	,376	,688
SEX * DIET * AS	67372905,041	2	33686452,521	2,340	,107
Error	690890230,21	48	14393546,463		
Total	1423186981,3	60			
Corrected Total	977957754,82	59			

a. R Squared = ,294 (Adjusted R Squared = ,132)

Pairwise Comparisons

Dependent Variable: vAT_CD8

SEX	AS	(I) DIET	(J) DIET	Mean Difference (I-J)	Std. Error	Sig.[a]	95% Confidence Interval for Difference[a] Lower Bound	Upper Bound
female	AS1	LFD	HFD	-3821,373	2190,399	,087	-8225,466	582,720
		HFD	LFD	3821,373	2190,399	,087	-582,720	8225,466
	AS2	LFD	HFD	-4693,432	2545,014	,071	-9810,525	423,661
		HFD	LFD	4693,432	2545,014	,071	-423,661	9810,525
	AS3	LFD	HFD	1977,748	2399,462	,414	-2846,694	6802,189
		HFD	LFD	-1977,748	2399,462	,414	-6802,189	2846,694
male	AS1	LFD	HFD	-73,220	2770,660	,979	-5644,006	5497,565
		HFD	LFD	73,220	2770,660	,979	-5497,565	5644,006
	AS2	LFD	HFD	4,532	2399,462	,999	-4819,910	4828,974
		HFD	LFD	-4,532	2399,462	,999	-4828,974	4819,910
	AS3	LFD	HFD	-2735,256	2297,310	,240	-7354,308	1883,796
		HFD	LFD	2735,256	2297,310	,240	-1883,796	7354,308

Based on estimated marginal means

a. Adjustment for multiple comparisons: Bonferroni.

scAT

Tests of Between-Subjects Effects

Dependent Variable: scAT_CD8

Source	Type III Sum of Squares	df	Mean Square	F	Sig.
Corrected Model	21811811,26[a]	11	1982891,933	2,067	,042
Intercept	88941783,462	1	88941783,462	92,728	<,001
SEX	6645468,635	1	6645468,635	6,928	,011
DIET	5247374,449	1	5247374,449	5,471	,024
AS	3999552,736	2	1999776,368	2,085	,136
SEX * DIET	814264,644	1	814264,644	,849	,362
SEX * AS	2180423,068	2	1090211,534	1,137	,330
DIET * AS	2310443,581	2	1155221,790	1,204	,309
SEX * DIET * AS	753958,269	2	376979,134	,393	,677
Error	45081017,513	47	959170,585		
Total	160419455,72	59			
Corrected Total	66892828,775	58			

a. R Squared = ,326 (Adjusted R Squared = ,168)

Pairwise Comparisons

Dependent Variable: scAT_CD8

SEX	AS	(I) DIET	(J) DIET	Mean Difference (I-J)	Std. Error	Sig.[a]	95% Confidence Interval for Difference[a]	
							Lower Bound	Upper Bound
female	AS1	LFD	HFD	-1036,667	593,040	,087	-2229,708	156,375
		HFD	LFD	1036,667	593,040	,087	-156,375	2229,708
	AS2	LFD	HFD	-296,000	619,410	,635	-1542,091	950,091
		HFD	LFD	296,000	619,410	,635	-950,091	1542,091
	AS3	LFD	HFD	-1174,800	619,410	,064	-2420,891	71,291
		HFD	LFD	1174,800	619,410	,064	-71,291	2420,891
male	AS1	LFD	HFD	59,256	619,410	,924	-1186,836	1305,347
		HFD	LFD	-59,256	619,410	,924	-1305,347	1186,836
	AS2	LFD	HFD	-43,350	656,983	,948	-1365,029	1278,329
		HFD	LFD	43,350	656,983	,948	-1278,329	1365,029
	AS3	LFD	HFD	-1106,150	656,983	,099	-2427,829	215,529
		HFD	LFD	1106,150	656,983	,099	-215,529	2427,829

Based on estimated marginal means

a. Adjustment for multiple comparisons: Bonferroni.

Th1 (CXCR3+CCR6-)

vAT

Tests of Between-Subjects Effects

Dependent Variable: vAT_Th1

Source	Type III Sum of Squares	df	Mean Square	F	Sig.
Corrected Model	6783456,604[a]	11	616677,873	1,695	,103
Intercept	8852382,541	1	8852382,541	24,331	<,001
SEX	1825507,167	1	1825507,167	5,017	,030
DIET	207098,489	1	207098,489	,569	,454
AS	2287452,227	2	1143726,113	3,144	,052
SEX * DIET	5920,946	1	5920,946	,016	,899
SEX * AS	1665628,137	2	832814,068	2,289	,112
DIET * AS	54614,720	2	27307,360	,075	,928
SEX * DIET * AS	653662,560	2	326831,280	,898	,414
Error	17463903,141	48	363831,315		
Total	33529868,452	60			
Corrected Total	24247359,746	59			

a. R Squared = ,280 (Adjusted R Squared = ,115)

Pairwise Comparisons

Dependent Variable: vAT_Th1

SEX	AS	(I) DIET	(J) DIET	Mean Difference (I-J)	Std. Error	Sig.[a]	95% Confidence Interval for Difference[a] Lower Bound	Upper Bound
female	AS1	LFD	HFD	-55,400	365,246	,880	-789,777	678,977
		HFD	LFD	55,400	365,246	,880	-678,977	789,777
	AS2	LFD	HFD	-466,241	381,487	,228	-1233,272	300,791
		HFD	LFD	466,241	381,487	,228	-300,791	1233,272
	AS3	LFD	HFD	103,849	381,487	,787	-663,183	870,880
		HFD	LFD	-103,849	381,487	,787	-870,880	663,183
male	AS1	LFD	HFD	-22,795	440,504	,959	-908,486	862,897
		HFD	LFD	22,795	440,504	,959	-862,897	908,486
	AS2	LFD	HFD	83,864	381,487	,827	-683,167	850,896
		HFD	LFD	-83,864	381,487	,827	-850,896	683,167
	AS3	LFD	HFD	-358,010	365,246	,332	-1092,387	376,367
		HFD	LFD	358,010	365,246	,332	-376,367	1092,387

Based on estimated marginal means

a. Adjustment for multiple comparisons: Bonferroni.

scAT

Tests of Between-Subjects Effects

Dependent Variable: scAT_Th1

Source	Type III Sum of Squares	df	Mean Square	F	Sig.
Corrected Model	5350033,505[a]	11	486366,682	1,980	,051
Intercept	3732901,336	1	3732901,336	15,198	<,001
SEX	289626,758	1	289626,758	1,179	,283
DIET	1045480,458	1	1045480,458	4,257	,044
AS	1160240,566	2	580120,283	2,362	,105
SEX * DIET	143960,821	1	143960,821	,586	,448
SEX * AS	1508546,630	2	754273,315	3,071	,055
DIET * AS	616083,197	2	308041,599	1,254	,294
SEX * DIET * AS	631302,195	2	315651,097	1,285	,286
Error	12035291,368	49	245618,191		
Total	21091988,740	61			
Corrected Total	17385324,873	60			

a. R Squared = ,308 (Adjusted R Squared = ,152)

Pairwise Comparisons

Dependent Variable: scAT_Th1

SEX	AS	(I) DIET	(J) DIET	Mean Difference (I-J)	Std. Error	Sig.[b]	95% Confidence Interval for Difference[b] Lower Bound	Upper Bound
female	AS1	LFD	HFD	-108,547	300,100	,719	-711,620	494,527
		HFD	LFD	108,547	300,100	,719	-494,527	711,620
	AS2	LFD	HFD	-48,740	313,444	,877	-678,630	581,150
		HFD	LFD	48,740	313,444	,877	-581,150	678,630
	AS3	LFD	HFD	-921,060*	313,444	,005	-1550,950	-291,170
		HFD	LFD	921,060*	313,444	,005	291,170	1550,950
male	AS1	LFD	HFD	-271,445	313,444	,391	-901,335	358,444
		HFD	LFD	271,445	313,444	,391	-358,444	901,335
	AS2	LFD	HFD	-67,800	313,444	,830	-697,690	562,090
		HFD	LFD	67,800	313,444	,830	-562,090	697,690
	AS3	LFD	HFD	-155,400	313,444	,622	-785,290	474,490
		HFD	LFD	155,400	313,444	,622	-474,490	785,290

Based on estimated marginal means

*. The mean difference is significant at the 0,05 level.

b. Adjustment for multiple comparisons: Bonferroni.

Th17 (CCR6+CXCR3-)

vAT

Tests of Between-Subjects Effects

Dependent Variable: vAT_TH17_cells

Source	Type III Sum of Squares	df	Mean Square	F	Sig.
Corrected Model	29052844,82[a]	11	2641167,711	2,148	,034
Intercept	39840530,699	1	39840530,699	32,404	<,001
SEX	7926826,000	1	7926826,000	6,447	,014
DIET	2819062,903	1	2819062,903	2,293	,136
AS	4207005,091	2	2103502,545	1,711	,191
SEX * DIET	25772,678	1	25772,678	,021	,885
SEX * AS	3637980,352	2	1818990,176	1,479	,238
DIET * AS	1317108,730	2	658554,365	,536	,589
SEX * DIET * AS	8171978,454	2	4085989,227	3,323	,044
Error	60245372,600	49	1229497,400		
Total	135364602,63	61			
Corrected Total	89298217,417	60			

a. R Squared = ,325 (Adjusted R Squared = ,174)

Pairwise Comparisons

Dependent Variable: vAT_TH17_cells

SEX	AS	(I) DIET	(J) DIET	Mean Difference (I-J)	Std. Error	Sig.[b]	95% Confidence Interval for Difference[b] Lower Bound	Upper Bound
female	AS1	LFD	HFD	-1678,500*	640,182	,012	-2964,993	-392,007
		HFD	LFD	1678,500*	640,182	,012	392,007	2964,993
	AS2	LFD	HFD	-299,440	701,284	,671	-1708,723	1109,843
		HFD	LFD	299,440	701,284	,671	-1109,843	1708,723
	AS3	LFD	HFD	793,600	701,284	,263	-615,683	2202,883
		HFD	LFD	-793,600	701,284	,263	-2202,883	615,683
male	AS1	LFD	HFD	-51,033	809,773	,950	-1678,333	1576,266
		HFD	LFD	51,033	809,773	,950	-1576,266	1678,333
	AS2	LFD	HFD	-182,800	701,284	,795	-1592,083	1226,483
		HFD	LFD	182,800	701,284	,795	-1226,483	1592,083
	AS3	LFD	HFD	-1200,933	671,428	,080	-2550,219	148,352
		HFD	LFD	1200,933	671,428	,080	-148,352	2550,219

Based on estimated marginal means

*. The mean difference is significant at the 0,05 level.

b. Adjustment for multiple comparisons: Bonferroni.

scAT

Tests of Between-Subjects Effects

Dependent Variable: scAT_Th17

Source	Type III Sum of Squares	df	Mean Square	F	Sig.
Corrected Model	3984057,412[a]	11	362187,037	2,417	,019
Intercept	5733799,730	1	5733799,730	38,262	<,001
SEX	1155708,051	1	1155708,051	7,712	,008
DIET	796407,191	1	796407,191	5,314	,026
AS	90887,297	2	45443,649	,303	,740
SEX * DIET	541821,355	1	541821,355	3,616	,064
SEX * AS	594644,393	2	297322,197	1,984	,149
DIET * AS	193933,181	2	96966,590	,647	,528
SEX * DIET * AS	219551,355	2	109775,678	,733	,486
Error	6743548,896	45	149856,642		
Total	17663737,980	57			
Corrected Total	10727606,308	56			

a. R Squared = ,371 (Adjusted R Squared = ,218)

Pairwise Comparisons

Dependent Variable: scAT_Th17

SEX	AS	(I) DIET	(J) DIET	Mean Difference (I-J)	Std. Error	Sig.[b]	95% Confidence Interval for Difference[b]	
							Lower Bound	Upper Bound
female	AS1	LFD	HFD	-751,900*	234,409	,002	-1224,023	-279,777
		HFD	LFD	751,900*	234,409	,002	279,777	1224,023
	AS2	LFD	HFD	-228,320	244,832	,356	-721,437	264,797
		HFD	LFD	228,320	244,832	,356	-264,797	721,437
	AS3	LFD	HFD	-332,720	244,832	,181	-825,837	160,397
		HFD	LFD	332,720	244,832	,181	-160,397	825,837
male	AS1	LFD	HFD	-2,638	273,730	,992	-553,960	548,683
		HFD	LFD	2,638	273,730	,992	-548,683	553,960
	AS2	LFD	HFD	40,800	244,832	,868	-452,317	533,917
		HFD	LFD	-40,800	244,832	,868	-533,917	452,317
	AS3	LFD	HFD	-164,200	282,708	,564	-733,602	405,202
		HFD	LFD	164,200	282,708	,564	-405,202	733,602

Based on estimated marginal means

*. The mean difference is significant at the 0,05 level.

b. Adjustment for multiple comparisons: Bonferroni.

TH1/17 (CCR6+CXCR3+)

vAT

Tests of Between-Subjects Effects

Dependent Variable: vAT_TH17.1

Source	Type III Sum of Squares	df	Mean Square	F	Sig.
Corrected Model	4463780,852[a]	11	405798,259	2,168	,033
Intercept	5266666,325	1	5266666,325	28,132	<,001
SEX	1309804,162	1	1309804,162	6,996	,011
DIET	704747,774	1	704747,774	3,764	,058
AS	794447,209	2	397223,604	2,122	,131
SEX * DIET	40128,752	1	40128,752	,214	,645
SEX * AS	164518,894	2	82259,447	,439	,647
DIET * AS	311838,596	2	155919,298	,833	,441
SEX * DIET * AS	1199291,455	2	599645,728	3,203	,049
Error	8986141,433	48	187211,280		
Total	19490494,774	60			
Corrected Total	13449922,285	59			

a. R Squared = ,332 (Adjusted R Squared = ,179)

Pairwise Comparisons

Dependent Variable: vAT_TH17.1

SEX	AS	(I) DIET	(J) DIET	Mean Difference (I-J)	Std. Error	Sig.[b]	95% Confidence Interval for Difference[b] Lower Bound	Upper Bound
female	AS1	LFD	HFD	-423,645	249,807	,096	-925,916	78,627
		HFD	LFD	423,645	249,807	,096	-78,627	925,916
	AS2	LFD	HFD	-694,555*	290,250	,021	-1278,142	-110,968
		HFD	LFD	694,555*	290,250	,021	110,968	1278,142
	AS3	LFD	HFD	298,900	273,650	,280	-251,311	849,111
		HFD	LFD	-298,900	273,650	,280	-849,111	251,311
male	AS1	LFD	HFD	-12,383	315,984	,969	-647,711	622,946
		HFD	LFD	12,383	315,984	,969	-622,946	647,711
	AS2	LFD	HFD	-103,853	273,650	,706	-654,064	446,358
		HFD	LFD	103,853	273,650	,706	-446,358	654,064
	AS3	LFD	HFD	-387,386	262,000	,146	-914,173	139,401
		HFD	LFD	387,386	262,000	,146	-139,401	914,173

Based on estimated marginal means

*. The mean difference is significant at the 0,05 level.

b. Adjustment for multiple comparisons: Bonferroni.

scAT

Tests of Between-Subjects Effects

Dependent Variable: scAT_Th17.1

Source	Type III Sum of Squares	df	Mean Square	F	Sig.
Corrected Model	679113,846[a]	11	61737,622	2,100	,038
Intercept	1272728,075	1	1272728,075	43,289	<,001
SEX	48634,521	1	48634,521	1,654	,204
DIET	245576,834	1	245576,834	8,353	,006
AS	162138,423	2	81069,212	2,757	,073
SEX * DIET	3389,877	1	3389,877	,115	,736
SEX * AS	177210,589	2	88605,294	3,014	,058
DIET * AS	38004,740	2	19002,370	,646	,528
SEX * DIET * AS	8516,460	2	4258,230	,145	,866
Error	1440630,824	49	29400,629		
Total	3412993,359	61			
Corrected Total	2119744,670	60			

a. R Squared = ,320 (Adjusted R Squared = ,168)

Pairwise Comparisons

Dependent Variable: scAT_Th17.1

SEX	AS	(I) DIET	(J) DIET	Mean Difference (I-J)	Std. Error	Sig.[a]	95% Confidence Interval for Difference[a] Lower Bound	Upper Bound
female	AS1	LFD	HFD	-150,767	103,828	,153	-359,417	57,883
		HFD	LFD	150,767	103,828	,153	-57,883	359,417
	AS2	LFD	HFD	-81,680	108,445	,455	-299,608	136,248
		HFD	LFD	81,680	108,445	,455	-136,248	299,608
	AS3	LFD	HFD	-193,520	108,445	,081	-411,448	24,408
		HFD	LFD	193,520	108,445	,081	-24,408	411,448
male	AS1	LFD	HFD	-54,597	108,445	,617	-272,525	163,331
		HFD	LFD	54,597	108,445	,617	-163,331	272,525
	AS2	LFD	HFD	-81,200	108,445	,458	-299,128	136,728
		HFD	LFD	81,200	108,445	,458	-136,728	299,128
	AS3	LFD	HFD	-200,600	108,445	,070	-418,528	17,328
		HFD	LFD	200,600	108,445	,070	-17,328	418,528

Based on estimated marginal means

a. Adjustment for multiple comparisons: Bonferroni.

Treg (CD25+)

vAT

Tests of Between-Subjects Effects

Dependent Variable: vAT_CD25

Source	Type III Sum of Squares	df	Mean Square	F	Sig.
Corrected Model	141294398,3[a]	11	12844945,301	2,235	,028
Intercept	240311026,68	1	240311026,68	41,810	<,001
SEX	38745,588	1	38745,588	,007	,935
DIET	40708434,075	1	40708434,075	7,082	,011
AS	37006157,694	2	18503078,847	3,219	,049
SEX * DIET	4085793,314	1	4085793,314	,711	,403
SEX * AS	14138951,953	2	7069475,976	1,230	,302
DIET * AS	19576234,488	2	9788117,244	1,703	,193
SEX * DIET * AS	18809400,510	2	9404700,255	1,636	,206
Error	270144260,30	47	5747750,219		
Total	688089872,15	59			
Corrected Total	411438658,61	58			

a. R Squared = ,343 (Adjusted R Squared = ,190)

Pairwise Comparisons

Dependent Variable: vAT_CD25

SEX	AS	(I) DIET	(J) DIET	Mean Difference (I-J)	Std. Error	Sig.[b]	95% Confidence Interval for Difference[b]	
							Lower Bound	Upper Bound
female	AS1	LFD	HFD	-330,677	1451,726	,821	-3251,173	2589,819
		HFD	LFD	330,677	1451,726	,821	-2589,819	3251,173
	AS2	LFD	HFD	-3671,690*	1608,256	,027	-6907,084	-436,296
		HFD	LFD	3671,690*	1608,256	,027	436,296	6907,084
	AS3	LFD	HFD	544,821	1516,278	,721	-2505,537	3595,180
		HFD	LFD	-544,821	1516,278	,721	-3595,180	2505,537
male	AS1	LFD	HFD	-264,297	1750,847	,881	-3786,548	3257,954
		HFD	LFD	264,297	1750,847	,881	-3257,954	3786,548
	AS2	LFD	HFD	-2747,368	1516,278	,076	-5797,727	302,991
		HFD	LFD	2747,368	1516,278	,076	-302,991	5797,727
	AS3	LFD	HFD	-3652,527*	1451,726	,015	-6573,023	-732,031
		HFD	LFD	3652,527*	1451,726	,015	732,031	6573,023

Based on estimated marginal means

*. The mean difference is significant at the 0,05 level.

b. Adjustment for multiple comparisons: Bonferroni.

scAT

Tests of Between-Subjects Effects

Dependent Variable: scAT_CD25

Source	Type III Sum of Squares	df	Mean Square	F	Sig.
Corrected Model	10285323,02[a]	11	935029,366	3,129	,003
Intercept	23362013,062	1	23362013,062	78,174	<,001
SEX	3541772,849	1	3541772,849	11,851	,001
DIET	4463261,473	1	4463261,473	14,935	<,001
AS	467424,158	2	233712,079	,782	,463
SEX * DIET	554581,787	1	554581,787	1,856	,179
SEX * AS	473323,650	2	236661,825	,792	,459
DIET * AS	570482,501	2	285241,250	,954	,392
SEX * DIET * AS	58536,717	2	29268,359	,098	,907
Error	14643532,419	49	298847,600		
Total	49049525,843	61			
Corrected Total	24928855,440	60			

a. R Squared = ,413 (Adjusted R Squared = ,281)

Pairwise Comparisons

Dependent Variable: scAT_CD25

SEX	AS	(I) DIET	(J) DIET	Mean Difference (I-J)	Std. Error	Sig.[b]	95% Confidence Interval for Difference[b]	
							Lower Bound	Upper Bound
female	AS1	LFD	HFD	-642,667	331,025	,058	-1307,886	22,553
		HFD	LFD	642,667	331,025	,058	-22,553	1307,886
	AS2	LFD	HFD	-594,400	345,744	,092	-1289,199	100,399
		HFD	LFD	594,400	345,744	,092	-100,399	1289,199
	AS3	LFD	HFD	-960,800*	345,744	,008	-1655,599	-266,001
		HFD	LFD	960,800*	345,744	,008	266,001	1655,599
male	AS1	LFD	HFD	-86,618	345,744	,803	-781,417	608,181
		HFD	LFD	86,618	345,744	,803	-608,181	781,417
	AS2	LFD	HFD	-303,400	345,744	,384	-998,199	391,399
		HFD	LFD	303,400	345,744	,384	-391,399	998,199
	AS3	LFD	HFD	-662,200	345,744	,061	-1356,999	32,599
		HFD	LFD	662,200	345,744	,061	-32,599	1356,999

Based on estimated marginal means

*. The mean difference is significant at the 0,05 level.

b. Adjustment for multiple comparisons: Bonferroni.

CD11b+

vAT

Tests of Between-Subjects Effects

Dependent Variable: vAT_CD11b

Source	Type III Sum of Squares	df	Mean Square	F	Sig.
Corrected Model	2,681E+11[a]	11	24373014456	3,676	<,001
Intercept	5,441E+11	1	5,441E+11	82,063	<,001
SEX	77125165837	1	77125165837	11,632	,001
DIET	66926176034	1	66926176034	10,094	,003
AS	23427794153	2	11713897076	1,767	,182
SEX * DIET	2601185556,2	1	2601185556,2	,392	,534
SEX * AS	33393911203	2	16696955601	2,518	,091
DIET * AS	1740405586,4	2	870202793,21	,131	,877
SEX * DIET * AS	55171003336	2	27585501668	4,160	,021
Error	3,249E+11	49	6630572307,9		
Total	1,230E+12	61			
Corrected Total	5,930E+11	60			

a. R Squared = ,452 (Adjusted R Squared = ,329)

Pairwise Comparisons

Dependent Variable: vAT_CD11b

SEX	AS	(I) DIET	(J) DIET	Mean Difference (I-J)	Std. Error	Sig.[b]	95% Confidence Interval for Difference[b] Lower Bound	Upper Bound
female	AS1	LFD	HFD	-100143,600*	47012,666	,038	-194619,090	-5668,110
		HFD	LFD	100143,600*	47012,666	,038	5668,110	194619,090
	AS2	LFD	HFD	-79477,840	51499,795	,129	-182970,553	24014,873
		HFD	LFD	79477,840	51499,795	,129	-24014,873	182970,553
	AS3	LFD	HFD	17625,002	51499,795	,734	-85867,711	121117,716
		HFD	LFD	-17625,002	51499,795	,734	-121117,716	85867,711
male	AS1	LFD	HFD	-4537,311	59466,841	,939	-124040,403	114965,781
		HFD	LFD	4537,311	59466,841	,939	-114965,781	124040,403
	AS2	LFD	HFD	-62623,800	51499,795	,230	-166116,513	40868,913
		HFD	LFD	62623,800	51499,795	,230	-40868,913	166116,513
	AS3	LFD	HFD	-174393,805*	49307,300	<,001	-273480,535	-75307,076
		HFD	LFD	174393,805*	49307,300	<,001	75307,076	273480,535

Based on estimated marginal means

*. The mean difference is significant at the 0,05 level.

b. Adjustment for multiple comparisons: Bonferroni.

scAT

Tests of Between-Subjects Effects

Dependent Variable: scAT_CDC11b

Source	Type III Sum of Squares	df	Mean Square	F	Sig.
Corrected Model	3,080E+11[a]	11	27998819734	4,345	<,001
Intercept	3,589E+11	1	3,589E+11	55,701	<,001
SEX	1,416E+11	1	1,416E+11	21,978	<,001
DIET	83583574731	1	83583574731	12,970	<,001
AS	5518517812,4	2	2759258906,2	,428	,654
SEX * DIET	45313313190	1	45313313190	7,032	,011
SEX * AS	11900554903	2	5950277451,4	,923	,404
DIET * AS	6085059972,0	2	3042529986,0	,472	,626
SEX * DIET * AS	1891619274,8	2	945809637,38	,147	,864
Error	3,222E+11	50	6444230603,0		
Total	1,003E+12	62			
Corrected Total	6,302E+11	61			

a. R Squared = ,489 (Adjusted R Squared = ,376)

Pairwise Comparisons

Dependent Variable: scAT_CDC11b

SEX	AS	(I) DIET	(J) DIET	Mean Difference (I-J)	Std. Error	Sig.[b]	95% Confidence Interval for Difference[b] Lower Bound	Upper Bound
female	AS1	LFD	HFD	-128671,633*	48609,511	,011	-226306,709	-31036,557
		HFD	LFD	128671,633*	48609,511	,011	31036,557	226306,709
	AS2	LFD	HFD	-90320,000	50770,978	,081	-192296,511	11656,511
		HFD	LFD	90320,000	50770,978	,081	-11656,511	192296,511
	AS3	LFD	HFD	-164400,000*	50770,978	,002	-266376,511	-62423,489
		HFD	LFD	164400,000*	50770,978	,002	62423,489	266376,511
male	AS1	LFD	HFD	-8761,699	48609,511	,858	-106396,775	88873,377
		HFD	LFD	8761,699	48609,511	,858	-88873,377	106396,775
	AS2	LFD	HFD	-13212,400	50770,978	,796	-115188,911	88764,111
		HFD	LFD	13212,400	50770,978	,796	-88764,111	115188,911
	AS3	LFD	HFD	-36254,400	50770,978	,478	-138230,911	65722,111
		HFD	LFD	36254,400	50770,978	,478	-65722,111	138230,911

Based on estimated marginal means

*. The mean difference is significant at the 0,05 level.

b. Adjustment for multiple comparisons: Bonferroni.

Spleen cell % statistics

CD19+

Tests of Between-Subjects Effects

Dependent Variable: spleen_percCD19

Source	Type III Sum of Squares	df	Mean Square	F	Sig.
Corrected Model	,677[a]	11	,062	4,404	<,001
Intercept	13,764	1	13,764	984,713	<,001
SEX	,423	1	,423	30,235	<,001
DIET	,001	1	,001	,095	,760
AS	,010	2	,005	,355	,703
SEX * DIET	,010	1	,010	,706	,405
SEX * AS	,237	2	,118	8,466	<,001
DIET * AS	,011	2	,005	,378	,687
SEX * DIET * AS	,010	2	,005	,347	,709
Error	,727	52	,014		
Total	15,278	64			
Corrected Total	1,404	63			

a. R Squared = ,482 (Adjusted R Squared = ,373)

Pairwise Comparisons

Dependent Variable: spleen_percCD19

SEX	AS	(I) DIET	(J) DIET	Mean Difference (I-J)	Std. Error	Sig.[a]	95% Confidence Interval for Difference[a]	
							Lower Bound	Upper Bound
female	AS1	LFD	HFD	-,057	,072	,432	-,200	,087
		HFD	LFD	,057	,072	,432	-,087	,200
	AS2	LFD	HFD	-,081	,075	,283	-,231	,069
		HFD	LFD	,081	,075	,283	-,069	,231
	AS3	LFD	HFD	,036	,072	,619	-,108	,179
		HFD	LFD	-,036	,072	,619	-,179	,108
male	AS1	LFD	HFD	,017	,072	,814	-,127	,161
		HFD	LFD	-,017	,072	,814	-,161	,127
	AS2	LFD	HFD	,013	,075	,858	-,137	,163
		HFD	LFD	-,013	,075	,858	-,163	,137
	AS3	LFD	HFD	,017	,072	,813	-,127	,161
		HFD	LFD	-,017	,072	,813	-,161	,127

Based on estimated marginal means

a. Adjustment for multiple comparisons: Bonferroni.

CD3+CD4+

Tests of Between-Subjects Effects

Dependent Variable: spleen_percCD4

Source	Type III Sum of Squares	df	Mean Square	F	Sig.
Corrected Model	,055[a]	11	,005	2,060	,041
Intercept	1,178	1	1,178	482,611	<,001
SEX	2,100E-5	1	2,100E-5	,009	,926
DIET	,000	1	,000	,171	,681
AS	,010	2	,005	2,096	,133
SEX * DIET	,000	1	,000	,127	,723
SEX * AS	,028	2	,014	5,643	,006
DIET * AS	,009	2	,005	1,906	,159
SEX * DIET * AS	,010	2	,005	1,947	,153
Error	,127	52	,002		
Total	1,385	64			
Corrected Total	,182	63			

a. R Squared = ,304 (Adjusted R Squared = ,156)

Pairwise Comparisons

Dependent Variable: spleen_percCD4

SEX	AS	(I) DIET	(J) DIET	Mean Difference (I-J)	Std. Error	Sig.[b]	95% Confidence Interval for Difference[b] Lower Bound	Upper Bound
female	AS1	LFD	HFD	,072*	,030	,020	,012	,132
		HFD	LFD	-,072*	,030	,020	-,132	-,012
	AS2	LFD	HFD	-,001	,031	,978	-,064	,062
		HFD	LFD	,001	,031	,978	-,062	,064
	AS3	LFD	HFD	-,042	,030	,163	-,102	,018
		HFD	LFD	,042	,030	,163	-,018	,102
male	AS1	LFD	HFD	-,004	,030	,889	-,064	,056
		HFD	LFD	,004	,030	,889	-,056	,064
	AS2	LFD	HFD	,013	,031	,678	-,050	,076
		HFD	LFD	-,013	,031	,678	-,076	,050
	AS3	LFD	HFD	-,007	,030	,822	-,067	,053
		HFD	LFD	,007	,030	,822	-,053	,067

Based on estimated marginal means

*. The mean difference is significant at the 0,05 level.

b. Adjustment for multiple comparisons: Bonferroni.

CD3+CD8+

Tests of Between-Subjects Effects

Dependent Variable: spleen_percCD8

Source	Type III Sum of Squares	df	Mean Square	F	Sig.
Corrected Model	,039[a]	11	,004	2,411	,017
Intercept	,459	1	,459	308,412	<,001
SEX	,000	1	,000	,161	,690
DIET	,000	1	,000	,243	,624
AS	,005	2	,003	1,709	,191
SEX * DIET	1,771E-5	1	1,771E-5	,012	,914
SEX * AS	,029	2	,014	9,623	<,001
DIET * AS	,003	2	,002	1,087	,345
SEX * DIET * AS	,003	2	,002	1,045	,359
Error	,077	52	,001		
Total	,584	64			
Corrected Total	,117	63			

a. R Squared = ,338 (Adjusted R Squared = ,198)

Pairwise Comparisons

Dependent Variable: spleen_percCD8

SEX	AS	(I) DIET	(J) DIET	Mean Difference (I-J)	Std. Error	Sig.[a]	95% Confidence Interval for Difference[a] Lower Bound	Upper Bound
female	AS1	LFD	HFD	,020	,023	,388	-,027	,067
		HFD	LFD	-,020	,023	,388	-,067	,027
	AS2	LFD	HFD	,005	,024	,845	-,044	,054
		HFD	LFD	-,005	,024	,845	-,054	,044
	AS3	LFD	HFD	-,043	,023	,074	-,090	,004
		HFD	LFD	,043	,023	,074	-,004	,090
male	AS1	LFD	HFD	-,012	,023	,622	-,058	,035
		HFD	LFD	,012	,023	,622	-,035	,058
	AS2	LFD	HFD	,007	,024	,762	-,042	,056
		HFD	LFD	-,007	,024	,762	-,056	,042
	AS3	LFD	HFD	-,007	,023	,766	-,054	,040
		HFD	LFD	,007	,023	,766	-,040	,054

Based on estimated marginal means

a. Adjustment for multiple comparisons: Bonferroni.

CD11b+

Tests of Between-Subjects Effects

Dependent Variable: spleen_percCD11b

Source	Type III Sum of Squares	df	Mean Square	F	Sig.
Corrected Model	,016[a]	11	,001	2,870	,005
Intercept	,191	1	,191	385,970	<,001
SEX	,009	1	,009	17,225	<,001
DIET	,000	1	,000	,331	,567
AS	,001	2	,001	1,310	,279
SEX * DIET	,000	1	,000	,316	,576
SEX * AS	,001	2	,000	,562	,573
DIET * AS	,002	2	,001	2,045	,140
SEX * DIET * AS	,003	2	,001	2,927	,062
Error	,026	52	,000		
Total	,234	64			
Corrected Total	,041	63			

a. R Squared = ,378 (Adjusted R Squared = ,246)

Pairwise Comparisons

Dependent Variable: spleen_percCD11b

SEX	AS	(I) DIET	(J) DIET	Mean Difference (I-J)	Std. Error	Sig.[b]	95% Confidence Interval for Difference[b]	
							Lower Bound	Upper Bound
female	AS1	LFD	HFD	-,024	,013	,081	-,051	,003
		HFD	LFD	,024	,013	,081	-,003	,051
	AS2	LFD	HFD	,032*	,014	,025	,004	,061
		HFD	LFD	-,032*	,014	,025	-,061	-,004
	AS3	LFD	HFD	,011	,013	,432	-,016	,038
		HFD	LFD	-,011	,013	,432	-,038	,016
male	AS1	LFD	HFD	-,001	,013	,926	-,028	,026
		HFD	LFD	,001	,013	,926	-,026	,028
	AS2	LFD	HFD	-,011	,014	,458	-,039	,018
		HFD	LFD	,011	,014	,458	-,018	,039
	AS3	LFD	HFD	,012	,013	,377	-,015	,039
		HFD	LFD	-,012	,013	,377	-,039	,015

Based on estimated marginal means

*. The mean difference is significant at the 0,05 level.

b. Adjustment for multiple comparisons: Bonferroni.

Th1 (CXCR3+CCR6-)

Tests of Between-Subjects Effects

Dependent Variable: spleen_percTh1

Source	Type III Sum of Squares	df	Mean Square	F	Sig.
Corrected Model	,004[a]	11	,000	3,304	,002
Intercept	,032	1	,032	302,505	<,001
SEX	,001	1	,001	7,238	,010
DIET	8,325E-5	1	8,325E-5	,782	,381
AS	,001	2	,000	4,423	,017
SEX * DIET	5,312E-6	1	5,312E-6	,050	,824
SEX * AS	,001	2	,001	6,927	,002
DIET * AS	,001	2	,000	2,579	,086
SEX * DIET * AS	,000	2	,000	1,274	,288
Error	,006	52	,000		
Total	,042	64			
Corrected Total	,009	63			

a. R Squared = ,411 (Adjusted R Squared = ,287)

Pairwise Comparisons

Dependent Variable: spleen_percTh1

SEX	AS	(I) DIET	(J) DIET	Mean Difference (I-J)	Std. Error	Sig.[b]	95% Confidence Interval for Difference[b]	
							Lower Bound	Upper Bound
female	AS1	LFD	HFD	,010	,006	,123	-,003	,022
		HFD	LFD	-,010	,006	,123	-,022	,003
	AS2	LFD	HFD	-,005	,007	,436	-,018	,008
		HFD	LFD	,005	,007	,436	-,008	,018
	AS3	LFD	HFD	-,013*	,006	,038	-,026	-,001
		HFD	LFD	,013*	,006	,038	,001	,026
male	AS1	LFD	HFD	-,001	,006	,923	-,013	,012
		HFD	LFD	,001	,006	,923	-,012	,013
	AS2	LFD	HFD	,001	,007	,838	-,012	,014
		HFD	LFD	-,001	,007	,838	-,014	,012
	AS3	LFD	HFD	-,006	,006	,352	-,018	,007
		HFD	LFD	,006	,006	,352	-,007	,018

Based on estimated marginal means

*. The mean difference is significant at the 0,05 level.

b. Adjustment for multiple comparisons: Bonferroni.

Th17 (CCR6+CXCR3-)

Tests of Between-Subjects Effects

Dependent Variable: spleen_percTh17

Source	Type III Sum of Squares	df	Mean Square	F	Sig.
Corrected Model	2,015E-5[a]	11	1,832E-6	2,256	,025
Intercept	,000	1	,000	229,674	<,001
SEX	1,413E-5	1	1,413E-5	17,392	<,001
DIET	3,913E-8	1	3,913E-8	,048	,827
AS	3,208E-6	2	1,604E-6	1,975	,149
SEX * DIET	4,269E-8	1	4,269E-8	,053	,820
SEX * AS	2,296E-6	2	1,148E-6	1,414	,253
DIET * AS	2,364E-7	2	1,182E-7	,146	,865
SEX * DIET * AS	1,003E-7	2	5,017E-8	,062	,940
Error	4,142E-5	51	8,122E-7		
Total	,000	63			
Corrected Total	6,157E-5	62			

a. R Squared = ,327 (Adjusted R Squared = ,182)

Pairwise Comparisons

Dependent Variable: spleen_percTh17

SEX	AS	(I) DIET	(J) DIET	Mean Difference (I-J)	Std. Error	Sig.[a]	95% Confidence Interval for Difference[a]	
							Lower Bound	Upper Bound
female	AS1	LFD	HFD	,000	,001	,789	-,001	,001
		HFD	LFD	,000	,001	,789	-,001	,001
	AS2	LFD	HFD	,000	,001	,780	-,001	,001
		HFD	LFD	,000	,001	,780	-,001	,001
	AS3	LFD	HFD	2,000E-5	,001	,972	-,001	,001
		HFD	LFD	-2,000E-5	,001	,972	-,001	,001
male	AS1	LFD	HFD	9,667E-5	,001	,860	-,001	,001
		HFD	LFD	-9,667E-5	,001	,860	-,001	,001
	AS2	LFD	HFD	,000	,001	,861	-,001	,001
		HFD	LFD	,000	,001	,861	-,001	,001
	AS3	LFD	HFD	,000	,001	,581	-,001	,001
		HFD	LFD	,000	,001	,581	-,001	,001

Based on estimated marginal means

a. Adjustment for multiple comparisons: Bonferroni.

Th1/17 (CCR6+CXCR3+)

Tests of Between-Subjects Effects

Dependent Variable: spleen_percTH17_1

Source	Type III Sum of Squares	df	Mean Square	F	Sig.
Corrected Model	3,394E-5[a]	11	3,085E-6	1,783	,081
Intercept	,000	1	,000	97,241	<,001
SEX	6,837E-6	1	6,837E-6	3,951	,052
DIET	7,882E-7	1	7,882E-7	,456	,503
AS	6,955E-6	2	3,478E-6	2,010	,144
SEX * DIET	5,242E-7	1	5,242E-7	,303	,584
SEX * AS	1,282E-5	2	6,411E-6	3,705	,031
DIET * AS	8,477E-9	2	4,239E-9	,002	,998
SEX * DIET * AS	4,301E-6	2	2,150E-6	1,243	,297
Error	8,998E-5	52	1,730E-6		
Total	,000	64			
Corrected Total	,000	63			

a. R Squared = ,274 (Adjusted R Squared = ,120)

Pairwise Comparisons

Dependent Variable: spleen_percTH17_1

SEX	AS	(I) DIET	(J) DIET	Mean Difference (I-J)	Std. Error	Sig.[a]	95% Confidence Interval for Difference[a] Lower Bound	Upper Bound
female	AS1	LFD	HFD	,000	,001	,755	-,001	,002
		HFD	LFD	,000	,001	,755	-,002	,001
	AS2	LFD	HFD	,000	,001	,848	-,002	,002
		HFD	LFD	,000	,001	,848	-,002	,002
	AS3	LFD	HFD	,001	,001	,164	,000	,003
		HFD	LFD	-,001	,001	,164	-,003	,000
male	AS1	LFD	HFD	,000	,001	,768	-,001	,002
		HFD	LFD	,000	,001	,768	-,002	,001
	AS2	LFD	HFD	,001	,001	,519	-,001	,002
		HFD	LFD	-,001	,001	,519	-,002	,001
	AS3	LFD	HFD	-,001	,001	,416	-,002	,001
		HFD	LFD	,001	,001	,416	-,001	,002

Based on estimated marginal means

a. Adjustment for multiple comparisons: Bonferroni.

Treg (CD25+)

Tests of Between-Subjects Effects

Dependent Variable: spleen_percCD25

Source	Type III Sum of Squares	df	Mean Square	F	Sig.
Corrected Model	,000[a]	11	3,713E-5	3,141	,003
Intercept	,003	1	,003	247,675	<,001
SEX	,000	1	,000	19,932	<,001
DIET	4,324E-7	1	4,324E-7	,037	,849
AS	4,044E-5	2	2,022E-5	1,711	,191
SEX * DIET	1,598E-6	1	1,598E-6	,135	,715
SEX * AS	5,455E-5	2	2,728E-5	2,308	,110
DIET * AS	4,246E-5	2	2,123E-5	1,796	,176
SEX * DIET * AS	3,740E-5	2	1,870E-5	1,582	,215
Error	,001	52	1,182E-5		
Total	,004	64			
Corrected Total	,001	63			

a. R Squared = ,399 (Adjusted R Squared = ,272)

Pairwise Comparisons

Dependent Variable: spleen_percCD25

SEX	AS	(I) DIET	(J) DIET	Mean Difference (I-J)	Std. Error	Sig.[b]	95% Confidence Interval for Difference[b]	
							Lower Bound	Upper Bound
female	AS1	LFD	HFD	,005*	,002	,026	,001	,009
		HFD	LFD	-,005*	,002	,026	-,009	-,001
	AS2	LFD	HFD	-,003	,002	,230	-,007	,002
		HFD	LFD	,003	,002	,230	-,002	,007
	AS3	LFD	HFD	-,001	,002	,743	-,005	,003
		HFD	LFD	,001	,002	,743	-,003	,005
male	AS1	LFD	HFD	,000	,002	,952	-,004	,004
		HFD	LFD	,000	,002	,952	-,004	,004
	AS2	LFD	HFD	,000	,002	,927	-,004	,005
		HFD	LFD	,000	,002	,927	-,005	,004
	AS3	LFD	HFD	-,001	,002	,708	-,005	,003
		HFD	LFD	,001	,002	,708	-,003	,005

Based on estimated marginal means

*. The mean difference is significant at the 0,05 level.

b. Adjustment for multiple comparisons: Bonferroni.

ASC specking

CD11b+

vAT

Tests of Between-Subjects Effects

Dependent Variable: ASC_vAT_CD11b

Source	Type III Sum of Squares	df	Mean Square	F	Sig.
Corrected Model	,382[a]	11	,035	9,358	<,001
Intercept	5,818	1	5,818	1569,011	<,001
SEX	,158	1	,158	42,739	<,001
DIET	,041	1	,041	10,982	,002
AS	,043	2	,021	5,789	,005
SEX * DIET	,083	1	,083	22,515	<,001
SEX * AS	,044	2	,022	5,907	,005
DIET * AS	,004	2	,002	,587	,560
SEX * DIET * AS	,003	2	,001	,400	,673
Error	,193	52	,004		
Total	6,614	64			
Corrected Total	,575	63			

a. R Squared = ,664 (Adjusted R Squared = ,593)

Pairwise Comparisons

Dependent Variable: ASC_vAT_CD11b

SEX	AS	(I) DIET	(J) DIET	Mean Difference (I-J)	Std. Error	Sig.[b]	95% Confidence Interval for Difference[b] Lower Bound	Upper Bound
female	AS1	LFD	HFD	-,014	,035	,692	-,085	,057
		HFD	LFD	,014	,035	,692	-,057	,085
	AS2	LFD	HFD	,036	,039	,360	-,042	,113
		HFD	LFD	-,036	,039	,360	-,113	,042
	AS3	LFD	HFD	,044	,039	,258	-,033	,121
		HFD	LFD	-,044	,039	,258	-,121	,033
male	AS1	LFD	HFD	-,131*	,037	<,001	-,205	-,057
		HFD	LFD	,131*	,037	<,001	,057	,205
	AS2	LFD	HFD	-,101*	,039	,011	-,178	-,024
		HFD	LFD	,101*	,039	,011	,024	,178
	AS3	LFD	HFD	-,138*	,037	<,001	-,212	-,064
		HFD	LFD	,138*	,037	<,001	,064	,212

Based on estimated marginal means

*. The mean difference is significant at the 0,05 level.

b. Adjustment for multiple comparisons: Bonferroni.

scAT

Tests of Between-Subjects Effects

Dependent Variable: ASC_sAT_CD11b

Source	Type III Sum of Squares	df	Mean Square	F	Sig.
Corrected Model	,226[a]	11	,021	7,686	<,001
Intercept	4,348	1	4,348	1624,816	<,001
SEX	,126	1	,126	47,266	<,001
DIET	,017	1	,017	6,428	,015
AS	,014	2	,007	2,647	,082
SEX * DIET	,010	1	,010	3,711	,060
SEX * AS	,033	2	,016	6,157	,004
DIET * AS	,010	2	,005	1,889	,163
SEX * DIET * AS	,008	2	,004	1,450	,245
Error	,123	46	,003		
Total	5,026	58			
Corrected Total	,349	57			

a. R Squared = ,648 (Adjusted R Squared = ,563)

Pairwise Comparisons

Dependent Variable: ASC_sAT_CD11b

SEX	AS	(I) DIET	(J) DIET	Mean Difference (I-J)	Std. Error	Sig.[b]	95% Confidence Interval for Difference[b]	
							Lower Bound	Upper Bound
female	AS1	LFD	HFD	-,020	,031	,530	-,083	,043
		HFD	LFD	,020	,031	,530	-,043	,083
	AS2	LFD	HFD	-,054	,033	,108	-,119	,012
		HFD	LFD	,054	,033	,108	-,012	,119
	AS3	LFD	HFD	,048	,035	,172	-,022	,118
		HFD	LFD	-,048	,035	,172	-,118	,022
male	AS1	LFD	HFD	-,102*	,035	,005	-,172	-,032
		HFD	LFD	,102*	,035	,005	,032	,172
	AS2	LFD	HFD	-,041	,033	,212	-,107	,024
		HFD	LFD	,041	,033	,212	-,024	,107
	AS3	LFD	HFD	-,042	,037	,259	-,115	,032
		HFD	LFD	,042	,037	,259	-,032	,115

Based on estimated marginal means

*. The mean difference is significant at the 0,05 level.

b. Adjustment for multiple comparisons: Bonferroni.

Spleen

Tests of Between-Subjects Effects

Dependent Variable: ASC_spleen_CD11b

Source	Type III Sum of Squares	df	Mean Square	F	Sig.
Corrected Model	,255[a]	11	,023	2,080	,039
Intercept	3,976	1	3,976	357,361	<,001
SEX	,018	1	,018	1,655	,204
DIET	,001	1	,001	,130	,719
AS	,057	2	,029	2,575	,086
SEX * DIET	,009	1	,009	,829	,367
SEX * AS	,094	2	,047	4,215	,020
DIET * AS	,081	2	,041	3,662	,033
SEX * DIET * AS	,005	2	,002	,209	,812
Error	,567	51	,011		
Total	4,735	63			
Corrected Total	,822	62			

a. R Squared = ,310 (Adjusted R Squared = ,161)

Pairwise Comparisons

Dependent Variable: ASC_spleen_CD11b

SEX	AS	(I) DIET	(J) DIET	Mean Difference (I-J)	Std. Error	Sig.[a]	95% Confidence Interval for Difference[a] Lower Bound	Upper Bound
female	AS1	LFD	HFD	-,111	,068	,110	-,248	,026
		HFD	LFD	,111	,068	,110	-,026	,248
	AS2	LFD	HFD	-,045	,067	,505	-,179	,089
		HFD	LFD	,045	,067	,505	-,089	,179
	AS3	LFD	HFD	,054	,064	,406	-,075	,182
		HFD	LFD	-,054	,064	,406	-,182	,075
male	AS1	LFD	HFD	-,037	,064	,565	-,165	,091
		HFD	LFD	,037	,064	,565	-,091	,165
	AS2	LFD	HFD	-,045	,067	,499	-,179	,089
		HFD	LFD	,045	,067	,499	-,089	,179
	AS3	LFD	HFD	,127	,064	,053	-,002	,255
		HFD	LFD	-,127	,064	,053	-,255	,002

Based on estimated marginal means

a. Adjustment for multiple comparisons: Bonferroni.

CD3+CD4+

vAT

Tests of Between-Subjects Effects

Dependent Variable: ASC_vAT_CD4

Source	Type III Sum of Squares	df	Mean Square	F	Sig.
Corrected Model	,154[a]	11	,014	2,724	,007
Intercept	2,025	1	2,025	393,025	<,001
SEX	,030	1	,030	5,912	,019
DIET	,012	1	,012	2,379	,129
AS	,005	2	,003	,495	,613
SEX * DIET	,004	1	,004	,720	,400
SEX * AS	,026	2	,013	2,511	,091
DIET * AS	,064	2	,032	6,200	,004
SEX * DIET * AS	,015	2	,008	1,473	,239
Error	,268	52	,005		
Total	2,471	64			
Corrected Total	,422	63			

a. R Squared = ,366 (Adjusted R Squared = ,231)

Pairwise Comparisons

Dependent Variable: ASC_vAT_CD4

SEX	AS	(I) DIET	(J) DIET	Mean Difference (I-J)	Std. Error	Sig.[b]	95% Confidence Interval for Difference[b]	
							Lower Bound	Upper Bound
female	AS1	LFD	HFD	-,071	,041	,093	-,154	,012
		HFD	LFD	,071	,041	,093	-,012	,154
	AS2	LFD	HFD	,045	,045	,330	-,046	,136
		HFD	LFD	-,045	,045	,330	-,136	,046
	AS3	LFD	HFD	,156*	,045	,001	,065	,247
		HFD	LFD	-,156*	,045	,001	-,247	-,065
male	AS1	LFD	HFD	-,022	,043	,617	-,109	,065
		HFD	LFD	,022	,043	,617	-,065	,109
	AS2	LFD	HFD	,002	,045	,969	-,089	,093
		HFD	LFD	-,002	,045	,969	-,093	,089
	AS3	LFD	HFD	,058	,043	,191	-,030	,145
		HFD	LFD	-,058	,043	,191	-,145	,030

Based on estimated marginal means

*. The mean difference is significant at the 0,05 level.

b. Adjustment for multiple comparisons: Bonferroni.

scAT

Tests of Between-Subjects Effects

Dependent Variable: ASC_scAT_CD4

Source	Type III Sum of Squares	df	Mean Square	F	Sig.
Corrected Model	,319[a]	11	,029	4,049	<,001
Intercept	2,216	1	2,216	309,448	<,001
SEX	,068	1	,068	9,446	,004
DIET	,002	1	,002	,295	,590
AS	,000	2	,000	,033	,968
SEX * DIET	,006	1	,006	,831	,367
SEX * AS	,129	2	,064	8,979	<,001
DIET * AS	,103	2	,051	7,163	,002
SEX * DIET * AS	,004	2	,002	,269	,765
Error	,329	46	,007		
Total	2,979	58			
Corrected Total	,648	57			

a. R Squared = ,492 (Adjusted R Squared = ,370)

Pairwise Comparisons

Dependent Variable: ASC_scAT_CD4

SEX	AS	(I) DIET	(J) DIET	Mean Difference (I-J)	Std. Error	Sig.[b]	95% Confidence Interval for Difference[b]	
							Lower Bound	Upper Bound
female	AS1	LFD	HFD	-,100	,051	,058	-,203	,003
		HFD	LFD	,100	,051	,058	-,003	,203
	AS2	LFD	HFD	,006	,054	,905	-,101	,114
		HFD	LFD	-,006	,054	,905	-,114	,101
	AS3	LFD	HFD	,068	,057	,234	-,046	,183
		HFD	LFD	-,068	,057	,234	-,183	,046
male	AS1	LFD	HFD	-,104	,057	,075	-,218	,011
		HFD	LFD	,104	,057	,075	-,011	,218
	AS2	LFD	HFD	,058	,054	,285	-,050	,166
		HFD	LFD	-,058	,054	,285	-,166	,050
	AS3	LFD	HFD	,144*	,060	,020	,024	,265
		HFD	LFD	-,144*	,060	,020	-,265	-,024

Based on estimated marginal means

*. The mean difference is significant at the 0,05 level.

b. Adjustment for multiple comparisons: Bonferroni.

Spleen

Tests of Between-Subjects Effects

Dependent Variable: ASC_spleen_CD4

Source	Type III Sum of Squares	df	Mean Square	F	Sig.
Corrected Model	,015[a]	11	,001	1,346	,228
Intercept	,250	1	,250	245,945	<,001
SEX	,006	1	,006	5,912	,019
DIET	,001	1	,001	1,362	,249
AS	,001	2	,000	,397	,674
SEX * DIET	,001	1	,001	,820	,370
SEX * AS	,005	2	,002	2,266	,114
DIET * AS	,002	2	,001	,883	,420
SEX * DIET * AS	,001	2	,001	,621	,542
Error	,051	50	,001		
Total	,317	62			
Corrected Total	,066	61			

a. R Squared = ,228 (Adjusted R Squared = ,059)

Pairwise Comparisons

Dependent Variable: ASC_spleen_CD4

SEX	AS	(I) DIET	(J) DIET	Mean Difference (I-J)	Std. Error	Sig.[a]	95% Confidence Interval for Difference[a]	
							Lower Bound	Upper Bound
female	AS1	LFD	HFD	-,036	,021	,087	-,077	,005
		HFD	LFD	,036	,021	,087	-,005	,077
	AS2	LFD	HFD	-,023	,021	,286	-,066	,020
		HFD	LFD	,023	,021	,286	-,020	,066
	AS3	LFD	HFD	,008	,019	,673	-,031	,047
		HFD	LFD	-,008	,019	,673	-,047	,031
male	AS1	LFD	HFD	-,010	,019	,597	-,049	,029
		HFD	LFD	,010	,019	,597	-,029	,049
	AS2	LFD	HFD	,006	,020	,762	-,034	,047
		HFD	LFD	-,006	,020	,762	-,047	,034
	AS3	LFD	HFD	-,002	,019	,906	-,041	,037
		HFD	LFD	,002	,019	,906	-,037	,041

Based on estimated marginal means

a. Adjustment for multiple comparisons: Bonferroni.

CD19+

vAT

Tests of Between-Subjects Effects

Dependent Variable: ASC_vAT_CD19

Source	Type III Sum of Squares	df	Mean Square	F	Sig.
Corrected Model	,617[a]	11	,056	2,145	,033
Intercept	13,458	1	13,458	514,650	<,001
SEX	,026	1	,026	,994	,323
DIET	,016	1	,016	,611	,438
AS	,103	2	,052	1,972	,149
SEX * DIET	,024	1	,024	,902	,347
SEX * AS	,413	2	,206	7,894	,001
DIET * AS	,045	2	,022	,856	,431
SEX * DIET * AS	1,625E-5	2	8,123E-6	,000	1,000
Error	1,360	52	,026		
Total	15,612	64			
Corrected Total	1,977	63			

a. R Squared = ,312 (Adjusted R Squared = ,167)

Pairwise Comparisons

Dependent Variable: ASC_vAT_CD19

SEX	AS	(I) DIET	(J) DIET	Mean Difference (I-J)	Std. Error	Sig.[a]	95% Confidence Interval for Difference[a]	
							Lower Bound	Upper Bound
female	AS1	LFD	HFD	,007	,093	,939	-,180	,195
		HFD	LFD	-,007	,093	,939	-,195	,180
	AS2	LFD	HFD	,066	,102	,522	-,139	,271
		HFD	LFD	-,066	,102	,522	-,271	,139
	AS3	LFD	HFD	,138	,102	,184	-,068	,343
		HFD	LFD	-,138	,102	,184	-,343	,068
male	AS1	LFD	HFD	-,067	,098	,495	-,264	,129
		HFD	LFD	,067	,098	,495	-,129	,264
	AS2	LFD	HFD	-,012	,102	,910	-,217	,194
		HFD	LFD	,012	,102	,910	-,194	,217
	AS3	LFD	HFD	,058	,098	,553	-,138	,255
		HFD	LFD	-,058	,098	,553	-,255	,138

Based on estimated marginal means

a. Adjustment for multiple comparisons: Bonferroni.

scAT

Tests of Between-Subjects Effects

Dependent Variable: ASC_scAT_CD19

Source	Type III Sum of Squares	df	Mean Square	F	Sig.
Corrected Model	,730[a]	11	,066	4,115	<,001
Intercept	11,264	1	11,264	698,601	<,001
SEX	,115	1	,115	7,118	,011
DIET	,007	1	,007	,460	,501
AS	,095	2	,047	2,933	,064
SEX * DIET	,002	1	,002	,135	,715
SEX * AS	,388	2	,194	12,023	<,001
DIET * AS	,117	2	,059	3,637	,035
SEX * DIET * AS	,032	2	,016	,977	,385
Error	,693	43	,016		
Total	13,640	55			
Corrected Total	1,423	54			

a. R Squared = ,513 (Adjusted R Squared = ,388)

Pairwise Comparisons

Dependent Variable: ASC_scAT_CD19

SEX	AS	(I) DIET	(J) DIET	Mean Difference (I-J)	Std. Error	Sig.[b]	95% Confidence Interval for Difference[b]	
							Lower Bound	Upper Bound
female	AS1	LFD	HFD	-,055	,077	,477	-,210	,100
		HFD	LFD	,055	,077	,477	-,100	,210
	AS2	LFD	HFD	-,072	,080	,376	-,234	,090
		HFD	LFD	,072	,080	,376	-,090	,234
	AS3	LFD	HFD	,094	,104	,370	-,115	,303
		HFD	LFD	-,094	,104	,370	-,303	,115
male	AS1	LFD	HFD	-,213*	,085	,016	-,385	-,041
		HFD	LFD	,213*	,085	,016	,041	,385
	AS2	LFD	HFD	-,018	,080	,828	-,180	,144
		HFD	LFD	,018	,080	,828	-,144	,180
	AS3	LFD	HFD	,120	,090	,190	-,061	,301
		HFD	LFD	-,120	,090	,190	-,301	,061

Based on estimated marginal means

*. The mean difference is significant at the 0,05 level.

b. Adjustment for multiple comparisons: Bonferroni.

Spleen

Tests of Between-Subjects Effects

Dependent Variable: ASC_spleen_CD19

Source	Type III Sum of Squares	df	Mean Square	F	Sig.
Corrected Model	,845[a]	11	,077	2,704	,008
Intercept	15,300	1	15,300	538,644	<,001
SEX	,060	1	,060	2,098	,154
DIET	,044	1	,044	1,566	,217
AS	,120	2	,060	2,105	,132
SEX * DIET	,026	1	,026	,911	,344
SEX * AS	,608	2	,304	10,706	<,001
DIET * AS	,029	2	,015	,517	,599
SEX * DIET * AS	,001	2	,000	,010	,990
Error	1,449	51	,028		
Total	17,398	63			
Corrected Total	2,294	62			

a. R Squared = ,368 (Adjusted R Squared = ,232)

Pairwise Comparisons

Dependent Variable: ASC_spleen_CD19

SEX	AS	(I) DIET	(J) DIET	Mean Difference (I-J)	Std. Error	Sig.[a]	95% Confidence Interval for Difference[a]	
							Lower Bound	Upper Bound
female	AS1	LFD	HFD	-,147	,109	,182	-,366	,071
		HFD	LFD	,147	,109	,182	-,071	,366
	AS2	LFD	HFD	-,068	,107	,526	-,282	,146
		HFD	LFD	,068	,107	,526	-,146	,282
	AS3	LFD	HFD	-,068	,102	,510	-,273	,137
		HFD	LFD	,068	,102	,510	-,137	,273
male	AS1	LFD	HFD	-,083	,102	,419	-,288	,122
		HFD	LFD	,083	,102	,419	-,122	,288
	AS2	LFD	HFD	,024	,107	,820	-,190	,238
		HFD	LFD	-,024	,107	,820	-,238	,190
	AS3	LFD	HFD	,021	,102	,841	-,184	,226
		HFD	LFD	-,021	,102	,841	-,226	,184

Based on estimated marginal means

a. Adjustment for multiple comparisons: Bonferroni.

CD3+CD8+

vAT

Tests of Between-Subjects Effects

Dependent Variable: ASC_vAT_CD8

Source	Type III Sum of Squares	df	Mean Square	F	Sig.
Corrected Model	,127[a]	11	,012	2,035	,044
Intercept	,695	1	,695	122,505	<,001
SEX	,012	1	,012	2,175	,147
DIET	,036	1	,036	6,366	,015
AS	,000	2	,000	,036	,964
SEX * DIET	,003	1	,003	,531	,470
SEX * AS	,009	2	,004	,760	,473
DIET * AS	,025	2	,012	2,165	,125
SEX * DIET * AS	,041	2	,021	3,646	,033
Error	,284	50	,006		
Total	1,121	62			
Corrected Total	,411	61			

a. R Squared = ,309 (Adjusted R Squared = ,157)

Pairwise Comparisons

Dependent Variable: ASC_vAT_CD8

SEX	AS	(I) DIET	(J) DIET	Mean Difference (I-J)	Std. Error	Sig.[b]	95% Confidence Interval for Difference[b] Lower Bound	Upper Bound
female	AS1	LFD	HFD	-,064	,043	,147	-,152	,023
		HFD	LFD	,064	,043	,147	-,023	,152
	AS2	LFD	HFD	,112*	,048	,023	,016	,207
		HFD	LFD	-,112*	,048	,023	-,207	-,016
	AS3	LFD	HFD	,140*	,048	,005	,045	,236
		HFD	LFD	-,140*	,048	,005	-,236	-,045
male	AS1	LFD	HFD	,055	,051	,284	-,047	,156
		HFD	LFD	-,055	,051	,284	-,156	,047
	AS2	LFD	HFD	,006	,048	,894	-,089	,102
		HFD	LFD	-,006	,048	,894	-,102	,089
	AS3	LFD	HFD	,042	,046	,357	-,049	,134
		HFD	LFD	-,042	,046	,357	-,134	,049

Based on estimated marginal means

*. The mean difference is significant at the 0,05 level.

b. Adjustment for multiple comparisons: Bonferroni.

scAT

Tests of Between-Subjects Effects

Dependent Variable: ASC_scAT_CD8

Source	Type III Sum of Squares	df	Mean Square	F	Sig.
Corrected Model	,137[a]	11	,012	2,102	,039
Intercept	,967	1	,967	163,233	<,001
SEX	,011	1	,011	1,838	,182
DIET	,001	1	,001	,156	,695
AS	,026	2	,013	2,169	,126
SEX * DIET	,001	1	,001	,168	,684
SEX * AS	,044	2	,022	3,708	,032
DIET * AS	,042	2	,021	3,564	,036
SEX * DIET * AS	,011	2	,005	,922	,405
Error	,272	46	,006		
Total	1,440	58			
Corrected Total	,409	57			

a. R Squared = ,334 (Adjusted R Squared = ,175)

Pairwise Comparisons

Dependent Variable: ASC_scAT_CD8

SEX	AS	(I) DIET	(J) DIET	Mean Difference (I-J)	Std. Error	Sig.[a]	95% Confidence Interval for Difference[a] Lower Bound	Upper Bound
female	AS1	LFD	HFD	-,085	,047	,075	-,179	,009
		HFD	LFD	,085	,047	,075	-,009	,179
	AS2	LFD	HFD	,069	,049	,164	-,029	,167
		HFD	LFD	-,069	,049	,164	-,167	,029
	AS3	LFD	HFD	,066	,052	,210	-,038	,170
		HFD	LFD	-,066	,052	,210	-,170	,038
male	AS1	LFD	HFD	-,047	,052	,371	-,151	,057
		HFD	LFD	,047	,052	,371	-,057	,151
	AS2	LFD	HFD	-,022	,049	,652	-,120	,076
		HFD	LFD	,022	,049	,652	-,076	,120
	AS3	LFD	HFD	,068	,054	,219	-,042	,177
		HFD	LFD	-,068	,054	,219	-,177	,042

Based on estimated marginal means

a. Adjustment for multiple comparisons: Bonferroni.

spleen

Tests of Between-Subjects Effects

Dependent Variable: ASC_spleen_CD8

Source	Type III Sum of Squares	df	Mean Square	F	Sig.
Corrected Model	,007[a]	11	,001	1,056	,414
Intercept	,058	1	,058	93,127	<,001
SEX	,001	1	,001	1,143	,290
DIET	1,810E-5	1	1,810E-5	,029	,865
AS	,001	2	,000	,534	,589
SEX * DIET	1,669E-5	1	1,669E-5	,027	,870
SEX * AS	,002	2	,001	1,989	,147
DIET * AS	,003	2	,001	2,186	,123
SEX * DIET * AS	,002	2	,001	1,275	,288
Error	,032	51	,001		
Total	,095	63			
Corrected Total	,039	62			

a. R Squared = ,185 (Adjusted R Squared = ,010)

Pairwise Comparisons

Dependent Variable: ASC_spleen_CD8

SEX	AS	(I) DIET	(J) DIET	Mean Difference (I-J)	Std. Error	Sig.[a]	95% Confidence Interval for Difference[a]	
							Lower Bound	Upper Bound
female	AS1	LFD	HFD	-,031	,016	,061	-,063	,001
		HFD	LFD	,031	,016	,061	-,001	,063
	AS2	LFD	HFD	,025	,016	,119	-,007	,057
		HFD	LFD	-,025	,016	,119	-,057	,007
	AS3	LFD	HFD	,006	,015	,693	-,024	,036
		HFD	LFD	-,006	,015	,693	-,036	,024
male	AS1	LFD	HFD	-,004	,015	,790	-,034	,026
		HFD	LFD	,004	,015	,790	-,026	,034
	AS2	LFD	HFD	,002	,016	,915	-,030	,033
		HFD	LFD	-,002	,016	,915	-,033	,030
	AS3	LFD	HFD	,009	,015	,567	-,022	,039
		HFD	LFD	-,009	,015	,567	-,039	,022

Based on estimated marginal means

a. Adjustment for multiple comparisons: Bonferroni.

Inflammatory cytokine z-score in blood plasma

Tests of Between-Subjects Effects

Dependent Variable: correct IL23, IL1a, IFNy, TNFa, MCP1, IL1b, GMCSF,IL17

Source	Type III Sum of Squares	df	Mean Square	F	Sig.
Corrected Model	8,720[a]	11	,793	2,400	,017
Intercept	,216	1	,216	,653	,423
SEX	3,965	1	3,965	12,005	,001
DIET	,271	1	,271	,822	,369
AS	2,193	2	1,096	3,319	,044
SEX * DIET	,647	1	,647	1,959	,168
SEX * AS	,432	2	,216	,654	,524
DIET * AS	,321	2	,161	,486	,618
SEX * DIET * AS	,846	2	,423	1,281	,286
Error	16,844	51	,330		
Total	25,649	63			
Corrected Total	25,564	62			

a. R Squared = ,341 (Adjusted R Squared = ,199)

Pairwise Comparisons

Dependent Variable: correct IL23, IL1a, IFNy, TNFa, MCP1, IL1b, GMCSF,IL17

SEX	AS	(I) DIET	(J) DIET	Mean Difference (I-J)	Std. Error	Sig.[a]	95% Confidence Interval for Difference[a]	
							Lower Bound	Upper Bound
female	AS1	LFD	HFD	-,400	,332	,234	-1,066	,266
		HFD	LFD	,400	,332	,234	-,266	1,066
	AS2	LFD	HFD	,123	,363	,736	-,607	,853
		HFD	LFD	-,123	,363	,736	-,853	,607
	AS3	LFD	HFD	,493	,348	,162	-,205	1,192
		HFD	LFD	-,493	,348	,162	-1,192	,205
male	AS1	LFD	HFD	-,246	,348	,483	-,945	,452
		HFD	LFD	,246	,348	,483	-,452	,945
	AS2	LFD	HFD	-,294	,386	,449	-1,068	,480
		HFD	LFD	,294	,386	,449	-,480	1,068
	AS3	LFD	HFD	-,472	,371	,209	-1,217	,272
		HFD	LFD	,472	,371	,209	-,272	1,217

Based on estimated marginal means

a. Adjustment for multiple comparisons: Bonferroni.

IL1b

Tests of Between-Subjects Effects

Dependent Variable: IL1b

Source	Type III Sum of Squares	df	Mean Square	F	Sig.
Corrected Model	10467,188[a]	11	951,563	2,673	,008
Intercept	19028,777	1	19028,777	53,459	<,001
SEX	3829,514	1	3829,514	10,759	,002
DIET	1527,783	1	1527,783	4,292	,043
AS	1483,442	2	741,721	2,084	,135
SEX * DIET	93,359	1	93,359	,262	,611
SEX * AS	500,171	2	250,085	,703	,500
DIET * AS	1283,076	2	641,538	1,802	,175
SEX * DIET * AS	1626,397	2	813,198	2,285	,112
Error	18509,407	52	355,950		
Total	49111,495	64			
Corrected Total	28976,595	63			

a. R Squared = ,361 (Adjusted R Squared = ,226)

Pairwise Comparisons

Dependent Variable: IL1b

SEX	AS	(I) DIET	(J) DIET	Mean Difference (I-J)	Std. Error	Sig.[b]	95% Confidence Interval for Difference[b]	
							Lower Bound	Upper Bound
female	AS1	LFD	HFD	-14,468	10,893	,190	-36,326	7,389
		HFD	LFD	14,468	10,893	,190	-7,389	36,326
	AS2	LFD	HFD	-35,020*	11,932	,005	-58,964	-11,076
		HFD	LFD	35,020*	11,932	,005	11,076	58,964
	AS3	LFD	HFD	12,657	11,424	,273	-10,268	35,581
		HFD	LFD	-12,657	11,424	,273	-35,581	10,268
male	AS1	LFD	HFD	-8,403	11,424	,465	-31,328	14,522
		HFD	LFD	8,403	11,424	,465	-14,522	31,328
	AS2	LFD	HFD	-5,410	11,932	,652	-29,354	18,534
		HFD	LFD	5,410	11,932	,652	-18,534	29,354
	AS3	LFD	HFD	-8,418	12,178	,492	-32,856	16,019
		HFD	LFD	8,418	12,178	,492	-16,019	32,856

Based on estimated marginal means

*. The mean difference is significant at the 0,05 level.

b. Adjustment for multiple comparisons: Bonferroni.

10 weeks dietary intervention

CD19+

vAT

Tests of Between-Subjects Effects

Dependent Variable: vAT_CD19

Source	Type III Sum of Squares	df	Mean Square	F	Sig.
Corrected Model	161463859,1[a]	3	53821286,382	23,335	<,001
Intercept	485860988,63	1	485860988,63	210,655	<,001
SEX	2851676,257	1	2851676,257	1,236	,280
DIET	159248626,73	1	159248626,73	69,045	<,001
SEX * DIET	2764619,147	1	2764619,147	1,199	,287
Error	43822270,192	19	2306435,273		
Total	725471728,89	23			
Corrected Total	205286129,34	22			

a. R Squared = ,787 (Adjusted R Squared = ,753)

Pairwise Comparisons

Dependent Variable: vAT_CD19

DIET	(I) SEX	(J) SEX	Mean Difference (I-J)	Std. Error	Sig.[a]	95% Confidence Interval for Difference[a]	
						Lower Bound	Upper Bound
LFD	female	male	1401,993	919,616	,144	-522,784	3326,771
	male	female	-1401,993	919,616	,144	-3326,771	522,784
HFD	female	male	10,867	876,819	,990	-1824,337	1846,070
	male	female	-10,867	876,819	,990	-1846,070	1824,337

Based on estimated marginal means

a. Adjustment for multiple comparisons: Bonferroni.

Pairwise Comparisons

Dependent Variable: vAT_CD19

SEX	(I) DIET	(J) DIET	Mean Difference (I-J)	Std. Error	Sig.[b]	95% Confidence Interval for Difference[b]	
						Lower Bound	Upper Bound
female	LFD	HFD	-4583,500*	876,819	<,001	-6418,703	-2748,297
	HFD	LFD	4583,500*	876,819	<,001	2748,297	6418,703
male	LFD	HFD	-5974,627*	919,616	<,001	-7899,404	-4049,849
	HFD	LFD	5974,627*	919,616	<,001	4049,849	7899,404

Based on estimated marginal means

*. The mean difference is significant at the 0,05 level.

b. Adjustment for multiple comparisons: Bonferroni.

scAT

Tests of Between-Subjects Effects

Dependent Variable: scAT_CD19

Source	Type III Sum of Squares	df	Mean Square	F	Sig.
Corrected Model	15187370,67[a]	3	5062456,889	13,355	<,001
Intercept	58383606,944	1	58383606,944	154,019	<,001
SEX	33363,679	1	33363,679	,088	,771
DIET	14290870,944	1	14290870,944	37,700	<,001
SEX * DIET	1762481,720	1	1762481,720	4,650	,047
Error	6065064,533	16	379066,533		
Total	87195028,000	20			
Corrected Total	21252435,200	19			

a. R Squared = ,715 (Adjusted R Squared = ,661)

Pairwise Comparisons

Dependent Variable: scAT_CD19

DIET	(I) SEX	(J) SEX	Mean Difference (I-J)	Std. Error	Sig.[a]	95% Confidence Interval for Difference[a] Lower Bound	Upper Bound
LFD	female	male	682,400	413,013	,118	-193,149	1557,949
	male	female	-682,400	413,013	,118	-1557,949	193,149
HFD	female	male	-517,333	372,815	,184	-1307,666	272,999
	male	female	517,333	372,815	,184	-272,999	1307,666

Based on estimated marginal means

a. Adjustment for multiple comparisons: Bonferroni.

Pairwise Comparisons

Dependent Variable: scAT_CD19

SEX	(I) DIET	(J) DIET	Mean Difference (I-J)	Std. Error	Sig.[b]	95% Confidence Interval for Difference[b] Lower Bound	Upper Bound
female	LFD	HFD	-1108,267*	372,815	,009	-1898,599	-317,934
	HFD	LFD	1108,267*	372,815	,009	317,934	1898,599
male	LFD	HFD	-2308,000*	413,013	<,001	-3183,549	-1432,451
	HFD	LFD	2308,000*	413,013	<,001	1432,451	3183,549

Based on estimated marginal means

*. The mean difference is significant at the 0,05 level.

b. Adjustment for multiple comparisons: Bonferroni.

CD3+CD4+

vAT

Tests of Between-Subjects Effects

Dependent Variable: vAT_CD4

Source	Type III Sum of Squares	df	Mean Square	F	Sig.
Corrected Model	182010748,8[a]	3	60670249,612	4,860	,011
Intercept	519575845,51	1	519575845,51	41,619	<,001
SEX	9421370,795	1	9421370,795	,755	,396
DIET	174948148,48	1	174948148,48	14,014	,001
SEX * DIET	92796,711	1	92796,711	,007	,932
Error	237197046,05	19	12484055,055		
Total	974412562,27	23			
Corrected Total	419207794,88	22			

a. R Squared = ,434 (Adjusted R Squared = ,345)

Pairwise Comparisons

Dependent Variable: vAT_CD4

DIET	(I) SEX	(J) SEX	Mean Difference (I-J)	Std. Error	Sig.[a]	95% Confidence Interval for Difference[a] Lower Bound	Upper Bound
LFD	female	male	1156,599	2139,506	,595	-3321,439	5634,637
	male	female	-1156,599	2139,506	,595	-5634,637	3321,439
HFD	female	male	1411,467	2039,939	,497	-2858,175	5681,108
	male	female	-1411,467	2039,939	,497	-5681,108	2858,175

Based on estimated marginal means

a. Adjustment for multiple comparisons: Bonferroni.

Pairwise Comparisons

Dependent Variable: vAT_CD4

SEX	(I) DIET	(J) DIET	Mean Difference (I-J)	Std. Error	Sig.[b]	95% Confidence Interval for Difference[b] Lower Bound	Upper Bound
female	LFD	HFD	-5660,600*	2039,939	,012	-9930,242	-1390,958
	HFD	LFD	5660,600*	2039,939	,012	1390,958	9930,242
male	LFD	HFD	-5405,732*	2139,506	,021	-9883,770	-927,694
	HFD	LFD	5405,732*	2139,506	,021	927,694	9883,770

Based on estimated marginal means

*. The mean difference is significant at the 0,05 level.

b. Adjustment for multiple comparisons: Bonferroni.

scAT

Tests of Between-Subjects Effects

Dependent Variable: scAT_CD4

Source	Type III Sum of Squares	df	Mean Square	F	Sig.
Corrected Model	20035249,62[a]	3	6678416,540	2,158	,131
Intercept	116208864,09	1	116208864,09	37,553	<,001
SEX	221548,545	1	221548,545	,072	,792
DIET	17401304,023	1	17401304,023	5,623	,030
SEX * DIET	1617915,571	1	1617915,571	,523	,479
Error	52607655,333	17	3094567,961		
Total	195767705,00	21			
Corrected Total	72642904,952	20			

a. R Squared = ,276 (Adjusted R Squared = ,148)

Pairwise Comparisons

Dependent Variable: scAT_CD4

DIET	(I) SEX	(J) SEX	Mean Difference (I-J)	Std. Error	Sig.[a]	95% Confidence Interval for Difference[a] Lower Bound	Upper Bound
LFD	female	male	-350,800	1112,577	,756	-2698,132	1996,532
	male	female	350,800	1112,577	,756	-1996,532	2698,132
HFD	female	male	762,933	1065,211	,484	-1484,466	3010,333
	male	female	-762,933	1065,211	,484	-3010,333	1484,466

Based on estimated marginal means

a. Adjustment for multiple comparisons: Bonferroni.

Pairwise Comparisons

Dependent Variable: scAT_CD4

SEX	(I) DIET	(J) DIET	Mean Difference (I-J)	Std. Error	Sig.[b]	95% Confidence Interval for Difference[b] Lower Bound	Upper Bound
female	LFD	HFD	-2383,133*	1065,211	,039	-4630,533	-135,734
	HFD	LFD	2383,133*	1065,211	,039	135,734	4630,533
male	LFD	HFD	-1269,400	1112,577	,270	-3616,732	1077,932
	HFD	LFD	1269,400	1112,577	,270	-1077,932	3616,732

Based on estimated marginal means

*. The mean difference is significant at the 0,05 level.

b. Adjustment for multiple comparisons: Bonferroni.

CD3+CD8+

vAT

Tests of Between-Subjects Effects

Dependent Variable: vAT_CD8

Source	Type III Sum of Squares	df	Mean Square	F	Sig.
Corrected Model	70270801,53[a]	3	23423600,509	2,885	,063
Intercept	144085601,08	1	144085601,08	17,747	<,001
SEX	7579607,527	1	7579607,527	,934	,346
DIET	61413411,735	1	61413411,735	7,564	,013
SEX * DIET	1685215,517	1	1685215,517	,208	,654
Error	154260878,84	19	8118993,623		
Total	379441140,73	23			
Corrected Total	224531680,36	22			

a. R Squared = ,313 (Adjusted R Squared = ,204)

Pairwise Comparisons

Dependent Variable: vAT_CD8

DIET	(I) SEX	(J) SEX	Mean Difference (I-J)	Std. Error	Sig.[a]	95% Confidence Interval for Difference[a] Lower Bound	Upper Bound
LFD	female	male	608,649	1725,388	,728	-3002,630	4219,928
	male	female	-608,649	1725,388	,728	-4219,928	3002,630
HFD	female	male	1694,767	1645,093	,316	-1748,453	5137,986
	male	female	-1694,767	1645,093	,316	-5137,986	1748,453

Based on estimated marginal means

a. Adjustment for multiple comparisons: Bonferroni.

Pairwise Comparisons

Dependent Variable: vAT_CD8

SEX	(I) DIET	(J) DIET	Mean Difference (I-J)	Std. Error	Sig.[b]	95% Confidence Interval for Difference[b] Lower Bound	Upper Bound
female	LFD	HFD	-3821,373*	1645,093	,031	-7264,593	-378,154
	HFD	LFD	3821,373*	1645,093	,031	378,154	7264,593
male	LFD	HFD	-2735,256	1725,388	,129	-6346,535	876,023
	HFD	LFD	2735,256	1725,388	,129	-876,023	6346,535

Based on estimated marginal means

*. The mean difference is significant at the 0,05 level.

b. Adjustment for multiple comparisons: Bonferroni.

scAT

Tests of Between-Subjects Effects

Dependent Variable: scAT_CD8

Source	Type III Sum of Squares	df	Mean Square	F	Sig.
Corrected Model	5690362,517[a]	3	1896787,506	2,803	,073
Intercept	21203946,613	1	21203946,613	31,329	<,001
SEX	28284,409	1	28284,409	,042	,841
DIET	5622444,817	1	5622444,817	8,307	,011
SEX * DIET	5911,755	1	5911,755	,009	,927
Error	10828991,283	16	676811,955		
Total	40285714,000	20			
Corrected Total	16519353,800	19			

a. R Squared = ,344 (Adjusted R Squared = ,222)

Pairwise Comparisons

Dependent Variable: scAT_CD8

DIET	(I) SEX	(J) SEX	Mean Difference (I-J)	Std. Error	Sig.[a]	95% Confidence Interval for Difference[a] Lower Bound	Upper Bound
LFD	female	male	-41,250	551,874	,941	-1211,172	1128,672
	male	female	41,250	551,874	,941	-1128,672	1211,172
HFD	female	male	-110,733	498,161	,827	-1166,787	945,321
	male	female	110,733	498,161	,827	-945,321	1166,787

Based on estimated marginal means

a. Adjustment for multiple comparisons: Bonferroni.

Pairwise Comparisons

Dependent Variable: scAT_CD8

SEX	(I) DIET	(J) DIET	Mean Difference (I-J)	Std. Error	Sig.[a]	95% Confidence Interval for Difference[a] Lower Bound	Upper Bound
female	LFD	HFD	-1036,667	498,161	,054	-2092,721	19,387
	HFD	LFD	1036,667	498,161	,054	-19,387	2092,721
male	LFD	HFD	-1106,150	551,874	,062	-2276,072	63,772
	HFD	LFD	1106,150	551,874	,062	-63,772	2276,072

Based on estimated marginal means

a. Adjustment for multiple comparisons: Bonferroni.

Th1 (CXCR3+CCR6-)

vAT

Tests of Between-Subjects Effects

Dependent Variable: vAT_Th1

Source	Type III Sum of Squares	df	Mean Square	F	Sig.
Corrected Model	401534,934[a]	3	133844,978	3,507	,037
Intercept	1040555,877	1	1040555,877	27,268	<,001
SEX	26919,000	1	26919,000	,705	,412
DIET	233056,227	1	233056,227	6,107	,024
SEX * DIET	124872,088	1	124872,088	3,272	,087
Error	686893,308	18	38160,739		
Total	2204781,616	22			
Corrected Total	1088428,242	21			

a. R Squared = ,369 (Adjusted R Squared = ,264)

Pairwise Comparisons

Dependent Variable: vAT_Th1

DIET	(I) SEX	(J) SEX	Mean Difference (I-J)	Std. Error	Sig.[a]	95% Confidence Interval for Difference[a]	
						Lower Bound	Upper Bound
LFD	female	male	81,054	118,289	,502	-167,461	329,570
	male	female	-81,054	118,289	,502	-329,570	167,461
HFD	female	male	-221,556	118,289	,077	-470,071	26,960
	male	female	221,556	118,289	,077	-26,960	470,071

Based on estimated marginal means

a. Adjustment for multiple comparisons: Bonferroni.

Pairwise Comparisons

Dependent Variable: vAT_Th1

SEX	(I) DIET	(J) DIET	Mean Difference (I-J)	Std. Error	Sig.[b]	95% Confidence Interval for Difference[b]	
						Lower Bound	Upper Bound
female	LFD	HFD	-55,400	118,289	,645	-303,916	193,116
	HFD	LFD	55,400	118,289	,645	-193,116	303,916
male	LFD	HFD	-358,010*	118,289	,007	-606,526	-109,494
	HFD	LFD	358,010*	118,289	,007	109,494	606,526

Based on estimated marginal means

*. The mean difference is significant at the 0,05 level.

b. Adjustment for multiple comparisons: Bonferroni.

scAT

Tests of Between-Subjects Effects

Dependent Variable: scAT_Th1

Source	Type III Sum of Squares	df	Mean Square	F	Sig.
Corrected Model	95392,688[a]	3	31797,563	2,363	,107
Intercept	382021,315	1	382021,315	28,389	<,001
SEX	4210,115	1	4210,115	,313	,583
DIET	90871,099	1	90871,099	6,753	,019
SEX * DIET	2863,350	1	2863,350	,213	,650
Error	228763,381	17	13456,669		
Total	719484,150	21			
Corrected Total	324156,070	20			

a. R Squared = ,294 (Adjusted R Squared = ,170)

Pairwise Comparisons

Dependent Variable: scAT_Th1

DIET	(I) SEX	(J) SEX	Mean Difference (I-J)	Std. Error	Sig.[a]	95% Confidence Interval for Difference[a] Lower Bound	Upper Bound
LFD	female	male	-4,980	73,367	,947	-159,770	149,810
	male	female	4,980	73,367	,947	-149,810	159,770
HFD	female	male	-51,833	70,243	,471	-200,034	96,367
	male	female	51,833	70,243	,471	-96,367	200,034

Based on estimated marginal means

a. Adjustment for multiple comparisons: Bonferroni.

Pairwise Comparisons

Dependent Variable: scAT_Th1

SEX	(I) DIET	(J) DIET	Mean Difference (I-J)	Std. Error	Sig.[b]	95% Confidence Interval for Difference[b] Lower Bound	Upper Bound
female	LFD	HFD	-108,547	70,243	,141	-256,747	39,654
	HFD	LFD	108,547	70,243	,141	-39,654	256,747
male	LFD	HFD	-155,400*	73,367	,049	-310,190	-,610
	HFD	LFD	155,400*	73,367	,049	,610	310,190

Based on estimated marginal means

*. The mean difference is significant at the 0,05 level.

b. Adjustment for multiple comparisons: Bonferroni.

Th17 (CCR6+CXCR3-)

vAT

Tests of Between-Subjects Effects

Dependent Variable: vAT_TH17_cells

Source	Type III Sum of Squares	df	Mean Square	F	Sig.
Corrected Model	13725985,44[a]	3	4575328,482	5,213	,009
Intercept	22258072,840	1	22258072,840	25,360	<,001
SEX	1653248,459	1	1653248,459	1,884	,186
DIET	11844480,459	1	11844480,459	13,495	,002
SEX * DIET	325814,173	1	325814,173	,371	,550
Error	16676184,033	19	877693,896		
Total	54548098,000	23			
Corrected Total	30402169,478	22			

a. R Squared = ,451 (Adjusted R Squared = ,365)

Pairwise Comparisons

Dependent Variable: vAT_TH17_cells

DIET	(I) SEX	(J) SEX	Mean Difference (I-J)	Std. Error	Sig.[a]	95% Confidence Interval for Difference[a]	
						Lower Bound	Upper Bound
LFD	female	male	299,100	567,293	,604	-888,257	1486,457
	male	female	-299,100	567,293	,604	-1486,457	888,257
HFD	female	male	776,667	540,892	,167	-355,434	1908,768
	male	female	-776,667	540,892	,167	-1908,768	355,434

Based on estimated marginal means

a. Adjustment for multiple comparisons: Bonferroni.

Pairwise Comparisons

Dependent Variable: vAT_TH17_cells

SEX	(I) DIET	(J) DIET	Mean Difference (I-J)	Std. Error	Sig.[b]	95% Confidence Interval for Difference[b]	
						Lower Bound	Upper Bound
female	LFD	HFD	-1678,500*	540,892	,006	-2810,601	-546,399
	HFD	LFD	1678,500*	540,892	,006	546,399	2810,601
male	LFD	HFD	-1200,933*	567,293	,048	-2388,291	-13,576
	HFD	LFD	1200,933*	567,293	,048	13,576	2388,291

Based on estimated marginal means

*. The mean difference is significant at the 0,05 level.

b. Adjustment for multiple comparisons: Bonferroni.

scAT

Tests of Between-Subjects Effects

Dependent Variable: scAT_Th17

Source	Type III Sum of Squares	df	Mean Square	F	Sig.
Corrected Model	2176457,658[a]	3	725485,886	2,150	,137
Intercept	2799468,100	1	2799468,100	8,297	,011
SEX	518169,344	1	518169,344	1,536	,234
DIET	932488,011	1	932488,011	2,764	,117
SEX * DIET	383768,100	1	383768,100	1,137	,303
Error	5061193,500	15	337412,900		
Total	11123044,000	19			
Corrected Total	7237651,158	18			

a. R Squared = ,301 (Adjusted R Squared = ,161)

Pairwise Comparisons

Dependent Variable: scAT_Th17

DIET	(I) SEX	(J) SEX	Mean Difference (I-J)	Std. Error	Sig.[a]	95% Confidence Interval for Difference[a]	
						Lower Bound	Upper Bound
LFD	female	male	47,600	424,209	,912	-856,581	951,781
	male	female	-47,600	424,209	,912	-951,781	856,581
HFD	female	male	635,300	351,736	,091	-114,407	1385,007
	male	female	-635,300	351,736	,091	-1385,007	114,407

Based on estimated marginal means

a. Adjustment for multiple comparisons: Bonferroni.

Pairwise Comparisons

Dependent Variable: scAT_Th17

SEX	(I) DIET	(J) DIET	Mean Difference (I-J)	Std. Error	Sig.[b]	95% Confidence Interval for Difference[b]	
						Lower Bound	Upper Bound
female	LFD	HFD	-751,900*	351,736	,049	-1501,607	-2,193
	HFD	LFD	751,900*	351,736	,049	2,193	1501,607
male	LFD	HFD	-164,200	424,209	,704	-1068,381	739,981
	HFD	LFD	164,200	424,209	,704	-739,981	1068,381

Based on estimated marginal means

*. The mean difference is significant at the 0,05 level.

b. Adjustment for multiple comparisons: Bonferroni.

Th1/17 (CCR6+CXCR3+)

vAT

Tests of Between-Subjects Effects

Dependent Variable: vAT_TH17.1

Source	Type III Sum of Squares	df	Mean Square	F	Sig.
Corrected Model	967405,486[a]	3	322468,495	3,916	,025
Intercept	1573135,492	1	1573135,492	19,105	<,001
SEX	33181,291	1	33181,291	,403	,533
DIET	939673,926	1	939673,926	11,412	,003
SEX * DIET	1878,128	1	1878,128	,023	,882
Error	1564529,285	19	82343,647		
Total	4238858,232	23			
Corrected Total	2531934,771	22			

a. R Squared = ,382 (Adjusted R Squared = ,285)

Pairwise Comparisons

Dependent Variable: vAT_TH17.1

DIET	(I) SEX	(J) SEX	Mean Difference (I-J)	Std. Error	Sig.[a]	95% Confidence Interval for Difference[a]	
						Lower Bound	Upper Bound
LFD	female	male	58,073	173,760	,742	-305,612	421,757
	male	female	-58,073	173,760	,742	-421,757	305,612
HFD	female	male	94,331	165,674	,576	-252,429	441,091
	male	female	-94,331	165,674	,576	-441,091	252,429

Based on estimated marginal means

a. Adjustment for multiple comparisons: Bonferroni.

Pairwise Comparisons

Dependent Variable: vAT_TH17.1

SEX	(I) DIET	(J) DIET	Mean Difference (I-J)	Std. Error	Sig.[b]	95% Confidence Interval for Difference[b]	
						Lower Bound	Upper Bound
female	LFD	HFD	-423,645*	165,674	,019	-770,405	-76,885
	HFD	LFD	423,645*	165,674	,019	76,885	770,405
male	LFD	HFD	-387,386*	173,760	,038	-751,071	-23,702
	HFD	LFD	387,386*	173,760	,038	23,702	751,071

Based on estimated marginal means

*. The mean difference is significant at the 0,05 level.

b. Adjustment for multiple comparisons: Bonferroni.

scAT

Tests of Between-Subjects Effects

Dependent Variable: scAT_Th17.1

Source	Type III Sum of Squares	df	Mean Square	F	Sig.
Corrected Model	163197,205[a]	3	54399,068	4,712	,014
Intercept	335766,436	1	335766,436	29,083	<,001
SEX	78,680	1	78,680	,007	,935
DIET	161032,871	1	161032,871	13,948	,002
SEX * DIET	3239,167	1	3239,167	,281	,603
Error	196264,033	17	11544,943		
Total	717227,000	21			
Corrected Total	359461,238	20			

a. R Squared = ,454 (Adjusted R Squared = ,358)

Pairwise Comparisons

Dependent Variable: scAT_Th17.1

DIET	(I) SEX	(J) SEX	Mean Difference (I-J)	Std. Error	Sig.[a]	95% Confidence Interval for Difference[a]	
						Lower Bound	Upper Bound
LFD	female	male	28,800	67,956	,677	-114,574	172,174
	male	female	-28,800	67,956	,677	-172,174	114,574
HFD	female	male	-21,033	65,063	,750	-158,303	116,237
	male	female	21,033	65,063	,750	-116,237	158,303

Based on estimated marginal means

a. Adjustment for multiple comparisons: Bonferroni.

Pairwise Comparisons

Dependent Variable: scAT_Th17.1

SEX	(I) DIET	(J) DIET	Mean Difference (I-J)	Std. Error	Sig.[b]	95% Confidence Interval for Difference[b]	
						Lower Bound	Upper Bound
female	LFD	HFD	-150,767*	65,063	,033	-288,037	-13,497
	HFD	LFD	150,767*	65,063	,033	13,497	288,037
male	LFD	HFD	-200,600*	67,956	,009	-343,974	-57,226
	HFD	LFD	200,600*	67,956	,009	57,226	343,974

Based on estimated marginal means

*. The mean difference is significant at the 0,05 level.

b. Adjustment for multiple comparisons: Bonferroni.

Treg (CD25+)
vAT

Tests of Between-Subjects Effects

Dependent Variable: vAT_CD25

Source	Type III Sum of Squares	df	Mean Square	F	Sig.
Corrected Model	49596258,01[a]	3	16532086,003	11,434	<,001
Intercept	108566059,98	1	108566059,98	75,090	<,001
SEX	9959203,864	1	9959203,864	6,888	,017
DIET	21635330,175	1	21635330,175	14,964	,001
SEX * DIET	15047301,031	1	15047301,031	10,407	,005
Error	26024700,370	18	1445816,687		
Total	192627122,36	22			
Corrected Total	75620958,378	21			

a. R Squared = ,656 (Adjusted R Squared = ,598)

Pairwise Comparisons

Dependent Variable: vAT_CD25

DIET	(I) SEX	(J) SEX	Mean Difference (I-J)	Std. Error	Sig.[b]	95% Confidence Interval for Difference[b]	
						Lower Bound	Upper Bound
LFD	female	male	309,683	728,102	,676	-1220,003	1839,369
	male	female	-309,683	728,102	,676	-1839,369	1220,003
HFD	female	male	-3012,167*	728,102	<,001	-4541,853	-1482,481
	male	female	3012,167*	728,102	<,001	1482,481	4541,853

Based on estimated marginal means

*. The mean difference is significant at the 0,05 level.

b. Adjustment for multiple comparisons: Bonferroni.

Pairwise Comparisons

Dependent Variable: vAT_CD25

SEX	(I) DIET	(J) DIET	Mean Difference (I-J)	Std. Error	Sig.[b]	95% Confidence Interval for Difference[b]	
						Lower Bound	Upper Bound
female	LFD	HFD	-330,677	728,102	,655	-1860,363	1199,009
	HFD	LFD	330,677	728,102	,655	-1199,009	1860,363
male	LFD	HFD	-3652,527*	728,102	<,001	-5182,213	-2122,841
	HFD	LFD	3652,527*	728,102	<,001	2122,841	5182,213

Based on estimated marginal means

*. The mean difference is significant at the 0,05 level.

b. Adjustment for multiple comparisons: Bonferroni.

scAT

Tests of Between-Subjects Effects

Dependent Variable: scAT_CD25

Source	Type III Sum of Squares	df	Mean Square	F	Sig.
Corrected Model	2880761,676[a]	3	960253,892	2,108	,137
Intercept	10101322,023	1	10101322,023	22,170	<,001
SEX	551878,545	1	551878,545	1,211	,286
DIET	2220883,067	1	2220883,067	4,874	,041
SEX * DIET	497,675	1	497,675	,001	,974
Error	7745616,133	17	455624,478		
Total	21475370,000	21			
Corrected Total	10626377,810	20			

a. R Squared = ,271 (Adjusted R Squared = ,142)

Pairwise Comparisons

Dependent Variable: scAT_CD25

DIET	(I) SEX	(J) SEX	Mean Difference (I-J)	Std. Error	Sig.[a]	95% Confidence Interval for Difference[a]	
						Lower Bound	Upper Bound
LFD	female	male	335,000	426,907	,443	-565,696	1235,696
	male	female	-335,000	426,907	,443	-1235,696	565,696
HFD	female	male	315,467	408,733	,451	-546,884	1177,817
	male	female	-315,467	408,733	,451	-1177,817	546,884

Based on estimated marginal means

a. Adjustment for multiple comparisons: Bonferroni.

Pairwise Comparisons

Dependent Variable: scAT_CD25

SEX	(I) DIET	(J) DIET	Mean Difference (I-J)	Std. Error	Sig.[a]	95% Confidence Interval for Difference[a]	
						Lower Bound	Upper Bound
female	LFD	HFD	-642,667	408,733	,134	-1505,017	219,684
	HFD	LFD	642,667	408,733	,134	-219,684	1505,017
male	LFD	HFD	-662,200	426,907	,139	-1562,896	238,496
	HFD	LFD	662,200	426,907	,139	-238,496	1562,896

Based on estimated marginal means

a. Adjustment for multiple comparisons: Bonferroni.

CD11b+

vAT

Tests of Between-Subjects Effects

Dependent Variable: vAT_CD11b

Source	Type III Sum of Squares	df	Mean Square	F	Sig.
Corrected Model	1,210E+11[a]	3	40341810855	7,927	,001
Intercept	3,885E+11	1	3,885E+11	76,339	<,001
SEX	11699720721	1	11699720721	2,299	,146
DIET	1,077E+11	1	1,077E+11	21,157	<,001
SEX * DIET	7875847131,5	1	7875847131,5	1,548	,229
Error	96696227123	19	5089275111,8		
Total	6,378E+11	23			
Corrected Total	2,177E+11	22			

a. R Squared = ,556 (Adjusted R Squared = ,486)

Pairwise Comparisons

Dependent Variable: vAT_CD11b

DIET	(I) SEX	(J) SEX	Mean Difference (I-J)	Std. Error	Sig.[a]	95% Confidence Interval for Difference[a]	
						Lower Bound	Upper Bound
LFD	female	male	82373,872	43198,004	,072	-8040,589	172788,333
	male	female	-82373,872	43198,004	,072	-172788,333	8040,589
HFD	female	male	8123,667	41187,681	,846	-78083,140	94330,473
	male	female	-8123,667	41187,681	,846	-94330,473	78083,140

Based on estimated marginal means

a. Adjustment for multiple comparisons: Bonferroni.

Pairwise Comparisons

Dependent Variable: vAT_CD11b

SEX	(I) DIET	(J) DIET	Mean Difference (I-J)	Std. Error	Sig.[b]	95% Confidence Interval for Difference[b]	
						Lower Bound	Upper Bound
female	LFD	HFD	-100143,600*	41187,681	,025	-186350,406	-13936,794
	HFD	LFD	100143,600*	41187,681	,025	13936,794	186350,406
male	LFD	HFD	-174393,805*	43198,004	<,001	-264808,267	-83979,344
	HFD	LFD	174393,805*	43198,004	<,001	83979,344	264808,267

Based on estimated marginal means

*. The mean difference is significant at the ,05 level.

b. Adjustment for multiple comparisons: Bonferroni.

scAT

Tests of Between-Subjects Effects

Dependent Variable: scAT_CDC11b

Source	Type III Sum of Squares	df	Mean Square	F	Sig.
Corrected Model	1,200E+11[a]	3	39994650107	8,169	,001
Intercept	1,615E+11	1	1,615E+11	32,982	<,001
SEX	64307359957	1	64307359957	13,136	,002
DIET	35479038875	1	35479038875	7,247	,015
SEX * DIET	11140363066	1	11140363066	2,276	,150
Error	83225759912	17	4895632936,0		
Total	3,875E+11	21			
Corrected Total	2,032E+11	20			

a. R Squared = ,590 (Adjusted R Squared = ,518)

Pairwise Comparisons

Dependent Variable: scAT_CDC11b

DIET	(I) SEX	(J) SEX	Mean Difference (I-J)	Std. Error	Sig.[b]	95% Confidence Interval for Difference[b] Lower Bound	Upper Bound
LFD	female	male	64812,000	44252,154	,161	-28551,885	158175,885
	male	female	-64812,000	44252,154	,161	-158175,885	28551,885
HFD	female	male	157229,233*	42368,212	,002	67840,119	246618,348
	male	female	-157229,233*	42368,212	,002	-246618,348	-67840,119

Based on estimated marginal means

*. The mean difference is significant at the ,05 level.

b. Adjustment for multiple comparisons: Bonferroni.

Pairwise Comparisons

Dependent Variable: scAT_CDC11b

SEX	(I) DIET	(J) DIET	Mean Difference (I-J)	Std. Error	Sig.[b]	95% Confidence Interval for Difference[b] Lower Bound	Upper Bound
female	LFD	HFD	-128671,633*	42368,212	,007	-218060,748	-39282,519
	HFD	LFD	128671,633*	42368,212	,007	39282,519	218060,748
male	LFD	HFD	-36254,400	44252,154	,424	-129618,285	57109,485
	HFD	LFD	36254,400	44252,154	,424	-57109,485	129618,285

Based on estimated marginal means

*. The mean difference is significant at the ,05 level.

b. Adjustment for multiple comparisons: Bonferroni.
