# Supplementary material for: Sex differentially affects pro-inflammatory cell subsets in adipose tissue depots in a diet induced obesity model
Source: Biol Sex Differ. 2024 Dec 18;15:105. doi: 10.1186/s13293-024-00677-1 (PMC11657622; doi:10.1186/s13293-024-00677-1)
Supplement: Supplementary file 2 — Supplementary Material 2. Table S1. Diet compositions. [file 13293_2024_677_MOESM2_ESM.docx]

### Table S1. Diet compositions.

|  |  | **HFD (D12492)** | **LFD (D12450J)** |
| --- | --- | --- | --- |
| **Protein** | Casein, Lactic, 30 Mesh | 200.00 g | 200.00 g |
|  | Cystine, L | 3.00 g | 3.00 g |
| **Carbohydrate** | Lodex 10 | 125.00 g | 125.00 g |
|  | Sucrose, fine granulated | 72.80 g | 72.80 g |
|  | Starch, Corn |  | 506.20 g |
| **Fiber** | Solka Floc, FCC200 | 50.00 g | 50.00 g |
| **Fat** | Lard | 245.00 g | 20.00 g |
|  | Soybean oil, USP | 25.00 g | 25.00 g |
| **Mineral** | S10026B | 50.00 g | 50.00 g |
| **Vitamin** | Choline Bitartrate | 2.00 g | 2.00 g |
|  | V10001C | 1.00 g | 1.00 g |
| **Dyes** |  | 0.05 g | 0.05 g |
