# Supplementary figures and images for: Sex differentially affects pro-inflammatory cell subsets in adipose tissue depots in a diet induced obesity model
Source: Biol Sex Differ. 2024 Dec 18;15:105. doi: 10.1186/s13293-024-00677-1 (PMC11657622; doi:10.1186/s13293-024-00677-1)

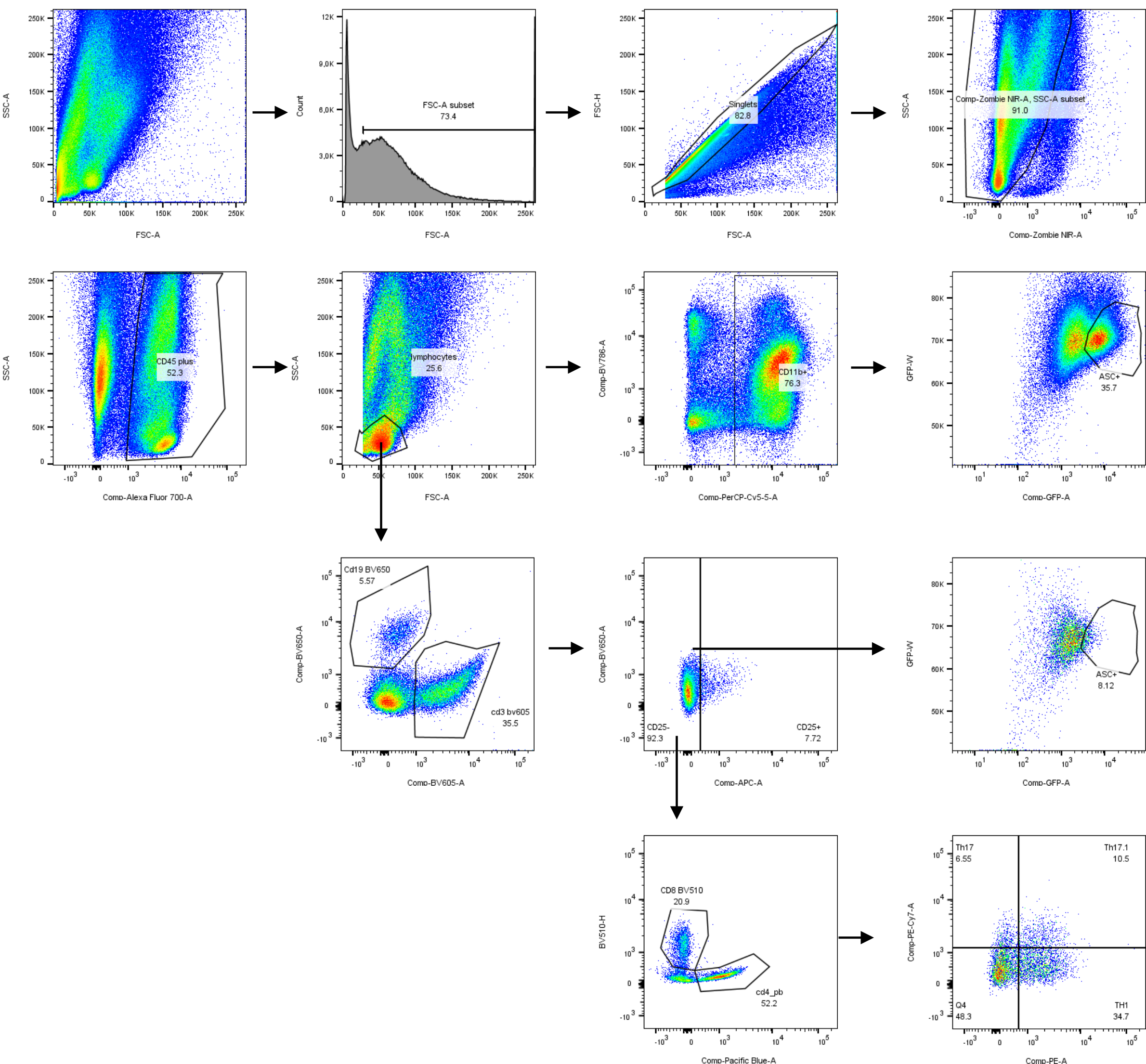

Supplement: Supplementary file 3 — Supplementary Material 3. Figure S1. Flow cytometry gating used for the whole experimental setup. Of all recorded cells a FSC-A subset corresponding to the MoxiTM counted cell number was used for singlet and live/dead gating. Lymphocytes (based on size) and myeloid cells (based on CD11b+) were gated from all CD45+ cells. CD19+ and CD3+ were used to determine lymphocyte lineage. To further gate T cells, CD25+ cells were excluded from the CD4+/CD8+ gating. The Th cell compartment was further divided into Th1 (CXCR3+CCR6-), Th17 (CCR6+CXCR3-) and Th1/17 (CXCR3+CCR6+). Of the major cell populations inflammasome activation was gated based on ASC specking. [file 13293_2024_677_MOESM3_ESM.png]

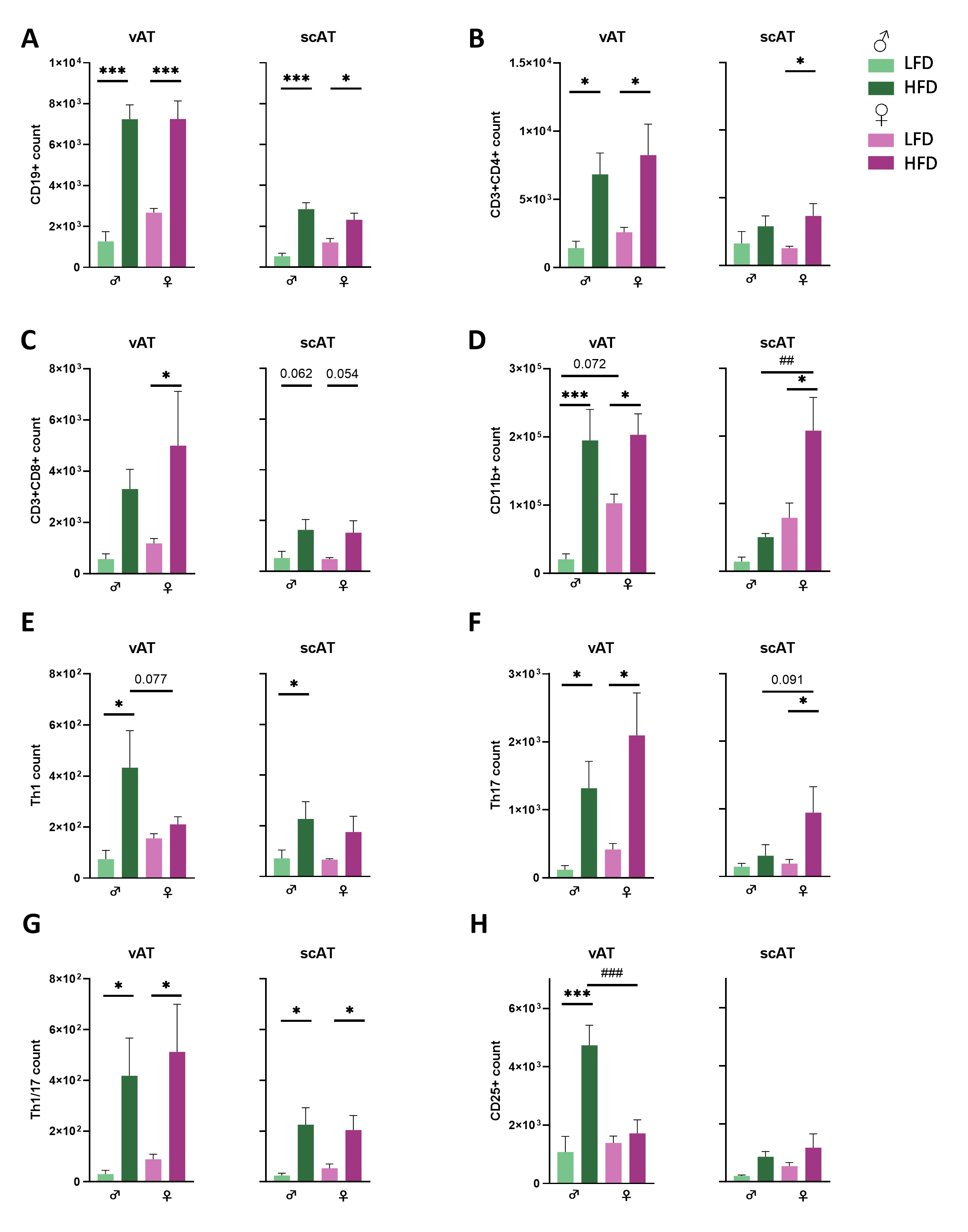

Supplement: Supplementary file 5 — Supplementary Material 5. Figure S3: Sex dimorphism is apparent at similar time on diet and age. Accumulation of B cells (A), CD3+CD4+ Th cells (B), CD3+CD8+ T cells (C), CD11b+ myeloid cells (D), Th1 (E), Th17 (F), Th1/17 (G) and CD25+ Treg (H) in vAT and scAT. Differences between male and female mice are seen in the scAT for CD11b+ myeloid cells and CD25+ Treg cells in vAT. (n=4-6/group, see Figure 2, 3, 4); p≤0.05*/#, p≤0.005**/##, p≤0.001***/###; *=changes based on diet; #=changes based on sex. [file 13293_2024_677_MOESM5_ESM.png]

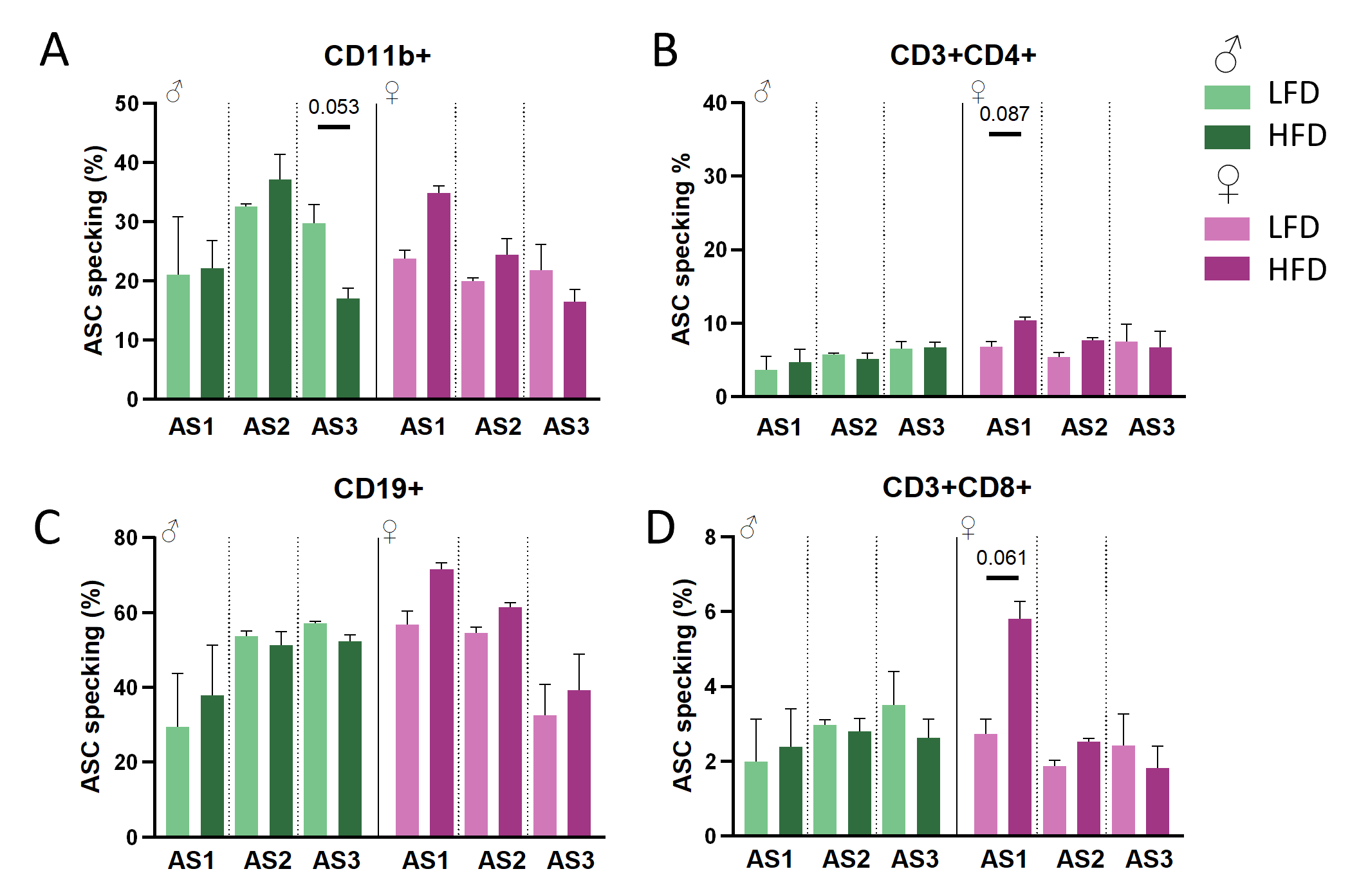

Supplement: Supplementary file 6 — Supplementary Material 6. Figure S4: In the spleen inflammasome activation in immune cells is not affected by the dietary intervention. % of ASC specking cells of CD11b+ myeloid cells (A), CD3+CD4+ Th cells (B), CD19+ B cells (C) and CD3+CD8+ T cells (D) of harvested splenocytes. In comparison to vAT and scAT, ASC specking due to inflammasome activation is lower in all immune cell subsets. (n=5-6/group, see Figure S2). [file 13293_2024_677_MOESM6_ESM.png]

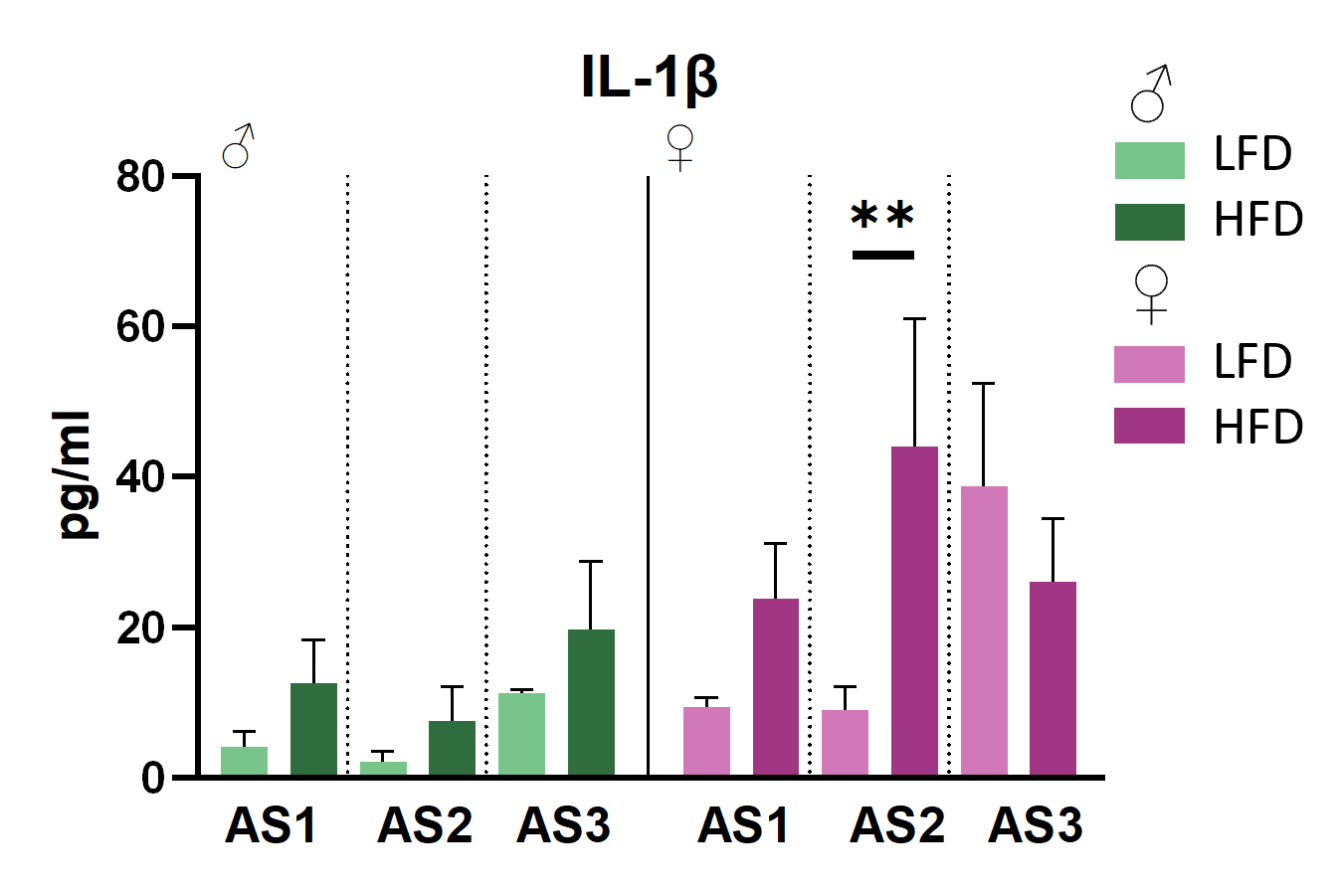

Supplement: Supplementary file 7 — Supplementary Material 7. Figure S5: IL-1β levels in blood plasma correlate with inflammasome activation in AT-derived immune cells of male mice, not in female mice. Concentration of IL-1β plasma levels at different AS in male and female mice after LFD or HFD. (n=6/♂ AS1 HFD, AS3 HFD ♀ AS1 HFD, LFD, AS3 LFD; n=5/♂ AS1 LFD, AS2 HFD, LFD, ♀ AS2 HFD, LFD, AS3 HFD; n=4/♂ AS3 LFD, ). [file 13293_2024_677_MOESM7_ESM.png]
